# Genetic analysis of vancomycin-variable *Enterococcus faecium* clinical isolates in Italy

**DOI:** 10.1007/s10096-024-04768-0

**Published:** 2024-01-31

**Authors:** Sonia Nina Coccitto, Marzia Cinthi, Serena Simoni, Antonella Pocognoli, Guido Zeni, Annarita Mazzariol, Gianluca Morroni, Marina Mingoia, Eleonora Giovanetti, Andrea Brenciani, Carla Vignaroli

**Affiliations:** 1 Department of Biomedical Sciences and Public Health, Polytechnic University of Marche, Ancona, Italy; 2 Department of Life and Environmental Sciences, Polytechnic University of Marche, Ancona, Italy; 3 Clinical Microbiology Laboratory, Azienda Ospedaliero-Universitaria “Ospedali Riuniti”, Ancona, Italy; 4 Department of Diagnostics and Public Health, Verona University, Verona, Italy

**Keywords:** *Enterococcus faecium*, Vancomycin variable enterococci, *vanA*, ST1478, *pstS*-null, Tn*1546*

## Abstract

**Purpose:**

To investigate the occurrence of vancomycin-variable enterococci (VVE) in a hospital in central Italy.

**Methods:**

vanA positive but vancomycin-susceptible *Enterococcus faecium* isolates (VVE-S) were characterized by antibiotic susceptibility tests, molecular typing (PFGE and MLST), and WGS approach. The reversion of VVE-S to a resistant phenotype was assessed by exposure to increasing vancomycin concentrations, and the revertant isolates were used in filter mating experiments. qPCR was used to analyze the plasmid copy number.

**Results:**

Eleven putative VVE-S were selected. WGS revealed two categories of *vanA* cluster plasmid located: the first type showed the lack of *vanR*, the deletion of *vanS*, and an intact *vanH*/*vanA*/*vanX* cluster; the second type was devoid of both *vanR* and *vanS* and showed a deletion of 544-bp at the 5′-end of the *vanH*. Strains (*n* = 7) carrying the first type of *vanA* cluster were considered VVE-S and were able to regain a resistance phenotype (VVE-R) in the presence of vancomycin, due to a 44-bp deletion in the promoter region of *vanH/vanA/vanX*, causing its constitutive expression. VVE-R strains were not able to transfer resistance by conjugation, and the resistance phenotype was unstable: after 11 days of growth without selective pressure, the revertants were still resistant but showed a lower vancomycin MIC. A higher plasmid copy number in the revertant strains was probably related to the resistance phenotype.

**Conclusion:**

We highlight the importance of VVE transition to VRE under vancomycin therapy resulting in a potential failure treatment. We also report the first-time identification of VVE-S isolates *pstS*-null belonging to ST1478.

**Supplementary Information:**

The online version contains supplementary material available at 10.1007/s10096-024-04768-0.

## Introduction

Vancomycin-resistant enterococci (VRE) are of great clinical significance worldwide [1]. Among all VRE species, vancomycin-resistant *Enterococcus faecium* (VREfm) is responsible for the majority of hospital infections and has been included in the list of priority pathogens against which the research and development of new antibiotics are urgently needed [2].

In *E. faecium*, vancomycin resistance is typically mediated by the *vanA* gene cluster carried by the Tn*1546* transposon. It consists of nine genes involved in transposition (*orf1* and *orf2*), signal transduction by a two-component system (*vanR* and *vanS*), vancomycin resistance (*vanH*, *vanA*, *vanX*, and *vanY*), and teicoplanin resistance (*vanZ*). The expression of the *vanH/vanA/vanX* cassette is controlled by the regulatory system *vanR*/*vanS*, where vanR is a response regulator and vanS is an integral membrane histidine kinase that recognizes the extracellular presence of vancomycin [3].

The Tn*1546* transposon (10.8 kb), located on the chromosome or on plasmids [4], is usually well conserved, despite it could be rearranged following several deletions or IS element insertion, both in intergenic regions and in coding sequences that determine genetic alterations in the *vanA* cluster [5].

A complete *vanH/vanA/vanX* cassette is necessary for the development of a vancomycin-resistant phenotype [6].

A particular variant of the VRE is represented by vancomycin-variable enterococci (VVE), i.e., vancomycin-susceptible enterococci with a *vanA* genotype (VVE-S), which can become resistant to vancomycin (VVE-R) upon exposure to vancomycin or teicoplanin [7]. The vancomycin resistance in VVE-S can occur from several mechanisms and result in the inducible or constitutive expression of *vanH*/*vanA*/*vanX* cassette [6, 8–12]. To date, VVE isolates have been reported in Canada [7, 8, 13–15], Norway [9, 11], Denmark [10, 16], South Korea [17], and very recently in India [18], Bangladesh [19], and Australia [6].

Interestingly, also *vanM*-carrying vancomycin-susceptible enterococci were detected in China, where tandem amplification of the *vanM* gene cluster was the primary mechanism for vancomycin resistance conversion [20].

VVE are capable of shifting from a glycopeptide-susceptible phenotype to a resistant phenotype during vancomycin therapy, thus limiting the success of treatment and representing an important source for vancomycin resistance genes.

In recent years, MLST non-typeable strains of vancomycin-resistant *E. faecium* that do not harbor the *pstS* gene (encoding a phosphate ATP-binding cassette transporter) have emerged [21]. These *pstS*-null sequence types (e.g., ST1421 and ST1424) have been reported in Australia, Denmark, Scotland, and South Korea [16, 21–23]. Very recently, a novel *pstS*-null ST1478 was also disseminated across acute care hospitals in Canada [24, 25].

The aim of this study was to survey the occurrence of clinical enterococci that were positive for *vanA*, but susceptible to vancomycin becoming from the “Ospedali Riuniti” hospital of Ancona to investigate (i) the clonal lineage and susceptibility patterns, (ii) the genetic context of the *vanA* gene cluster, (iii) their potential to revert to a vancomycin-resistant phenotype in vitro, and (iv) the transmission of the revertant *vanA* to an enterococcal recipient.

## Materials and methods

### Selection of VVE strains

From December 2021 to June 2022, 236 enterococci (*E. faecalis n* = 177 and *E. faecium n* = 59) were collected from different specimens obtained from hospitalized patients in several wards of the Ancona Regional Hospital. To prevent duplicate isolates, only one strain for each patient was included in the study. The isolates were identified by MALDI-TOF MS (Vitek MS, bioMerieux, France).

All isolates were tested by PCR for the presence of *vanA* and *vanB* genes, the most common glycopeptide resistance genes, as previously described [26].

The *vanA*-positive strains were tested for vancomycin susceptibility by standard broth microdilution, and only susceptible ones (VVE*Ita*-S) were further analyzed.

### Susceptibility testing

Susceptibilities to linezolid, chloramphenicol, ampicillin, ciprofloxacin, erythromycin, and tetracycline were performed by standard broth microdilution assays. The isolates were also tested for their susceptibility to tedizolid by E-test (Liofilchem, Roseto degli Abruzzi, Italy) according to the manufacturer’s instructions. Susceptibility tests were interpreted according to EUCAST (version v 13.0, www.eucast.org) and CLSI [27] breakpoints. *E. faecalis* ATCC 29212 was used as quality control.

### Typing experiments

Typing was performed by SmaI-PFGE and multi-locus sequence typing (MLST) assays. Macrorestriction with SmaI endonuclease (New England Biolabs, Beverly, MA) and pulsed-field gel electrophoresis (PFGE) analysis were performed as described elsewhere [28]. The banding pattern was analyzed using BioNumerics software and interpreted according to the criteria of Tenover et al. [29].

MLST was conducted using PCR and sequencing of the seven housekeeping genes. Allelic profiles and sequence types (STs) were assigned according to the database available on the MLST website (https://pubmlst.org/).

### Whole genome sequencing and bioinformatic analysis

Bacterial genomic DNA was extracted by the QIAcube automated extractor using the DNeasy PowerLyzer PowerSoil Kit, according to the manufacturer’s instructions (Qiagen, Germany). Extracted DNA was subjected to whole genome sequencing (WGS) by a hybrid method using both short-read sequencing (Illumina MiSeq platform) with a 2 × 150 bp paired-end approach and a long-read sequencing (MinION, Oxford Nanopore Technologies, Oxford, UK).

Hybrid assembly was performed with Unicycler v.0.4.8 (https://github.com/rrwick/Unicycler). In silico analysis of WGS data for identification of acquired antimicrobial resistance genes and virulence factors, the phylogenetic correlations, and plasmid replicon types were carried out using dedicated tools available at the Center for Genomic Epidemiology available at http://www.genomicepidemiology.org/ (ResFinder 4.1, VirulenceFinder 2.0, CSI Phylogeny 1.4 and PlasmidFinder 2.1) and by the BLAST site (https://blast.ncbi.nlm.nih.gov/Blast.cgi). The BPROM online tool (http://www.softberry.com/berry.phtml?topic=bprom&group=programs&subgroup=gfindb) was used to detect consensus promoter sequences.

### In vitro development of vancomycin resistance and its stability in VVE*Ita-*R

In order to assess the reversion of VVE*Ita*-S strains to the resistance phenotype under laboratory conditions, we tested strains by plating on agar supplemented with increasing concentrations of vancomycin (from 0.25 to 4 mg/L) as described previously [8]. Vancomycin-resistant revertant (VVE*Ita*-R) isolates were verified by MALDI-TOF, antimicrobial susceptibility testing, and WGS. The constitutive or inducible expression of vancomycin resistance was verified in MIC assays with or without 0.25 mg/L vancomycin induction.

Over time (for 15 days), vancomycin-resistance stability was evaluated by daily serial passages of VVE*Ita*-R isolate on antibiotic-free brain heart infusion agar (BHIA) (Oxoid, Basingstoke, UK) at 37 °C. After each overnight passage, 15 randomly chosen colonies were tested for susceptibility to vancomycin, and the DNA was extracted and screened by PCR for the presence of the *vanA* gene. Mutants were analyzed by SmaI-PFGE to confirm their relatedness with the respective VVE*Ita*-R isolates.

### qPCR assays

qPCR assays were performed to determine the copy number of the *vanA-*plasmid. DNA was extracted using the GenElute Bacterial Genomic DNA Kit (Sigma-Aldrich) from standardized broth cultures (10^8^ CFU/mL). qPCR reaction was performed in technical triplicate in a total volume of 20 µL containing 0.2 µM of each primer targeting the *vanA* gene, 10 µL of 2XRotor-Gene SYBR Green PCR master mix (Qiagen), and 2 µL of DNA. Water was used as a negative control. Cycling conditions were 95 °C for 5 min followed by 35 cycles of 94 °C for 10 s, 63 °C for 30 s of annealing, and 72 °C for 20 s. To quantify the copy number of the *vanA*-plasmid, a calibration curve was constructed using scalar dilutions (from 10^−5^ to 10^−9^ ng/reaction) of the *vanA* amplicon. Data were analyzed using Qiagen’s Rotor Gene Q Series software. Plasmid copies were calculated based on *vanA* amplicon size (196 bp) and the weight of 1 bp (1.095 × 10^−12^ ng) [30]. The results are reported as the average of three biological replicates in three qPCR assays ± standard deviation (SD).

### Mating experiments

Conjugative transfer of the *vanA* gene was assessed by filter mating experiments as previously described [31] using as a recipient *E. faecium* 64/3, a vancomycin-susceptible, and fusidic acid/rifampicin-resistant strain [32]. Transconjugants were selected on brain heart infusion agar BHIA plates containing vancomycin (4 mg/L), rifampicin, and fusidic acid (50 mg/L). Plates were incubated at 37 °C for 24–48 h and then examined for the presence of transconjugants. Conjugation frequencies were expressed as the ratio of cell number (CFU/mL) of transconjugants to the recipient. Transconjugants were evaluated for their susceptibility to vancomycin and tested by PCR for the presence of the *vanA* gene. SmaI-PFGE patterns were analyzed to confirm the genetic background of transconjugants.

### Nucleotide sequence accession numbers

The WGS data are available under the BioProject ID PRJNA993974. The nucleotide sequences of the 8 *vanA* plasmids have been deposited in GenBank under the following accession numbers: OR208591, OR234011, OR234012, OR234015, OR251469, OR251470, OR262468, and OR298096.

## Results and discussion

### Phenotypic characterization of VVE*Ita*-S

In this study, a collection of 236 clinical enterococci was analyzed for VVE detection. Eleven *E. faecium* strains (4.6% of all enterococcal isolates) were considered putative VVE (VVE*Ita*-S) being *vanA* positive and vancomycin susceptible. In our hospital, the frequency of putative VVE (19%) among the *E. faecium* clinical isolates (*n* = 59) was higher than those reported in other studies and surveys [9, 16, 18], although a progressive increase of the VVE prevalence has been recently reported in several countries [6, 15, 16]. The 11 putative VVE*Ita*-S were also tested for their susceptibility to ampicillin, ciprofloxacin, erythromycin, linezolid, tedizolid, and chloramphenicol, as summarized in Table 1. All the isolates were resistant to ampicillin and ciprofloxacin (MIC, > 128 mg/L) and ten to erythromycin (MIC, > 128 mg/L). All isolates were susceptible, besides to vancomycin, to linezolid (MIC, range 1–4 mg/L), tedizolid (MIC, range 0.5–1 mg/L), and chloramphenicol (MIC, range 8–16 mg/L).

**Table 1:** Distinctive features, susceptibility patterns, and molecular typing of the 11 vancomycin susceptible isolates were investigated

VVE*Ita*-S isolate	Isolation data			MIC (mg/L)								Vancomycin resistance gene	*pstS* gene	MLST sequence type	PFGE pulsotype
	Isolation date	Source	Ward	VAN	LZD	TZD	CHL	TET	ERY	CIP	AMP	*vanA*			
700907	Dec 21	Urine	Urology	1	2	0.5	16	0.5	> 128	> 128	> 128	+	−	ST1478	A
727475	Jan 22	Blood	Hepatology	2	4	0.5	8	0.5	> 128	> 128	> 128	+	−	ST1478	A
749325	Apr 22	Blood	Infectious diseases	0.5	1	1	8	0.5	> 128	> 128	> 128	+	−	ST1478	A
749286	Apr 22	Blood	Infectious diseases	0.5	1	0.5	8	32	> 128	> 128	> 128	+	−	ST1478	A
741160	Mar 22	Pus	Surgery	1	4	1	8	0.5	> 128	> 128	> 128	+	−	ST1478	A1
732558	Feb 22	Urine	Cardiology	2	2	1	8	0.25	> 128	> 128	> 128	+	+	ST80	B
733387	Feb 22	Urine	Surgery	1	2	0.5	8	0.25	> 128	> 128	> 128	+	+	ST80	B1
731980	Feb 22	Ascitic fluid	Hepatology	4	2	0.5	8	4	64	> 128	> 128	+	+	ST80	B2
755686	May 2022	Urine	Gastroenterology	2	2	1	8	128	> 128	> 128	> 128	+	+	ST80	C
742783	Mar 22	Abdominal drainage	ICU	1	2	1	8	0.5	> 128	> 128	> 128	+	+	ST117	D
735902	Feb 22	Peritoneal fluid	Surgery	1	2	0.5	8	0.25	> 128	> 128	> 128	+	+	ST789	E

*LZD*, linezolid; *TZD*, tedizolid; *CHL*, chloramphenicol; *VAN*, vancomycin; *TET*, tetracycline; *ERY*, erythromycin; *CIP*, ciprofloxacin, *AMP*, ampicillin

### Molecular analysis of VVE*Ita*-S strains

The 11 putative VVE*Ita*-S were typed by PFGE and MLST to establish their genetic relatedness (Table 1).

PFGE clustering showed two main clones: the first one included the 700,907, 727,475, 749,325, and 749,286 isolates exhibiting the same PFGE pattern (pulsotype A) and the closely related 741,160 isolate (A1 pulsotype). The second clone was represented by the 732,558 isolate showing the pulsotype B and the closely related 733,387 and 731,980 strains with pulsotypes B1 and B2, respectively. The 755,686, 742,783, and 735,902 isolates belonged to three different pulsotypes: C, D, and E, respectively (Figure S1).

By MLST five strains, associated to pulsotype A and A1 (700,907, 749,325, 749,286, 727,475, and 741,160), belonged to the newly reported *pstS*-null sequence type 1478 (ST1478). To the best of our knowledge, this is the first report of VVE-S included in this particular clonal lineage. Previously, VVE strains lacking the *pstS* gene have been identified only in the ST1421 clone [6, 10, 16]. To date, VRE ST1478 was only detected across Canadian hospitals by national surveillance [24, 25].

The isolates included in the B, B1, B2, and C pulsotypes belonged to ST80, whereas the 742,783 (pulsotype D) and 735,902 (pulsotype E) isolates were associated to ST117 and ST789, respectively (Table 1). Many of the more recently dominant *E. faecium* sequence types associated with nosocomial infection, such as ST80, ST789, and ST117, are derived from the well-known clonal complex CC17 [33]. Interestingly, the ST789 has been reported for clinical vancomycin-resistant *E. faecium* isolates in Algeria [34]. To date, most of the VVE described in the literature are included in the ST1421, ST203, and ST18 [6, 9–11, 14, 16], different from those found in this study (ST117 and ST80 (Table 1)), probably due to VVE*Ita*-S evolution from the most common VRE circulating clones in our hospital [26].

### Genome analysis of VVE*Ita*-S strains

On the basis of PFGE results, one strain for each pulsotype was subjected to WGS, resulting in a total of eight isolates sequenced (Table 2).

**Table 2:** Main features of the 7 VVE*Ita*-S strains and the *E. faecium* 741,160 subjected to WGS

VVE*Ita*-S isolate	PFGE pulsotype	MLST sequence type	Resistance genes	Virulence factor	Plasmid size (bp)	Rep family plasmid	Tn*1546* transposon	
							Mutated region of Tn*1546*	IS*Efm1* element
700907	A	ST1478	*msrC*, *erm*(B), *vanHAX*, *ant*(6)-la, *aph*(3′)-III, *aac*(6′)-li, *aac*(6′)-*aph*(2″), *dfrG*	*acm*, *efaAfm*, *hylEfm*, *espfm*	46.567 bp	RepA_N	vanR*, ∆*vanS*	+
741160	A1	ST1478	*msrC*, *erm*(B), *vanHAX*, *ant*(6)-la, *aph*(3′)-III, *aac*(6′)-li, *aac*(6′)-*aph*(2″), *dfrG*, *vanX*	*acm*, *efaAfm*, *hylEfm*, *espfm*	42.047 bp	RepA_N	vanR*, vanS*, ∆*vanH*	+
732558	B	ST80	*msrC*, *vanHAX*, *aac*(6′)-li	*acm*, *efaAfm*, *hylEfm*, *espfm*	45.236 bp	RepA_N	vanR*, ∆*vanS*	+
733387	B1	ST80	*msrC*, *erm*(B), *vanHAX*, *ant*(6)-la, *aph*(3′)-III, *aac*(6′)-li, *aac*(6′)-*aph*(2″)	*acm*, *efaAfm*, *hylEfm*, *espfm*	44.592 bp	RepA_N	vanR*, ∆*vanS*	+
731980	B2	ST80	*msrC*, *erm*(B), *vanHAX*, *ant*(6)-la, *aph*(3′)-III, *aac*(6′)-li, *aac*(6′)-*aph*(2″)	*acm*, *efaAfm*, *hylEfm*, *espfm*	47.689 bp	RepA_N	vanR*, ∆*vanS*	+
755686	C	ST80	*msrC*, *erm*(B), *vanHAX*, *ant*(6)-la, *aph*(3′)-III, *aac*(6′)-li, *aac*(6′)-*aph*(2″), *dfrG*, *tet*(L), *tet*(M)	*acm*, *efaAfm*, *hylEfm*, *espfm*	56.710 bp	RepA_N	vanR*, ∆*vanS*	−
742783	D	ST117	*msrC*, *erm*(B), *vanHAX*, *ant*(6)-la, *aph*(3′)-III, *aac*(6′)-li, *aac*(6′)-*aph*(2″), *tet*(M)	*acm*, *efaAfm*, *hylEfm*	46.438 bp	RepA_N	vanR*, ∆*vanS*	+
735902	E	ST789	*msrC*, *vanHAX*, *aac*(6′)-li, *tet*(M)	*acm*, *efaAfm*, *hylEfm*	50.385 bp	RepA_N	vanR*, ∆*vanS*	∆

^*^ stands for missed ORF; ∆ stands for truncated ORF

ResFinder analysis of the eight genomes revealed complex resistomes for the presence of several acquired antibiotic resistance genes (Table 2). All isolates shared, besides *vanH*/*vanA*/*vanX* cluster, *msrC* (resistance to macrolides and streptogramins A) and *aac*(6′)-Ii (resistance to aminoglycosides). The *erm*(B) (resistance to macrolides, lincosamides, and streptogramins A) and *ant*(6)-Ia, *aph*(3′)-III, and *aac*(6′)-*aph*(2″) (resistance to aminoglycosides) were identified in all but two isolates (732,558 and 735,902). Three out of eight genomes exhibited the *dfrG* (resistance to trimethoprim) and *tet*(M) (resistance to tetracycline) genes, and only the 755,686 genome showed the double combination *tet*(M)/*tet*(L).

The study of the virulome revealed that the eight isolates shared three acquired virulence genes: *acm* (collagen-binding protein), *efaAfm* (gelatinase), and hylEfm (hyaluronidase). The *espfm* (enterococcal surface protein biofilm associated to biofilm production) was identified in all but two isolates (742,783 and 735,902).

In silico analysis of WGS data showed that the 8 isolates exhibited different SNPs (from a minimum of 18 to a maximum of 3762), as detailed in Table S1. The clonal relatedness among strains is also shown in Figure S2.

An in-depth analysis of the 700,907 and 741,160 genomes, belonging to the *pstS*-null ST1478, clarified the absence of the housekeeping gene. Indeed, this gene was truncated by the insertion of a 2894 bp region, containing two ISL*3* family transposases, causing the loss of 503 bp of the *pstS* gene. However, despite the missing *pstS* in these two genomes, sequencing analysis revealed a *pstS* homologue within a *pst* operon (also referred to as *pstS2*), which is thought to be the actual *pstS* housekeeping gene in *E. faecium* [22].

Moreover, Lemonidis et al. suggested that a Tn*5801*-like transposon, usually carrying the *tet*(M) gene, is frequently found in *vanA*-type *pstS*-null strains [22]. However, neither the *tet*(M) gene nor the Tn*5801* transposon was identified in the 700,907 and 741,160 genomes.

### Analysis of the *vanA* cluster

**Fig. 1:**
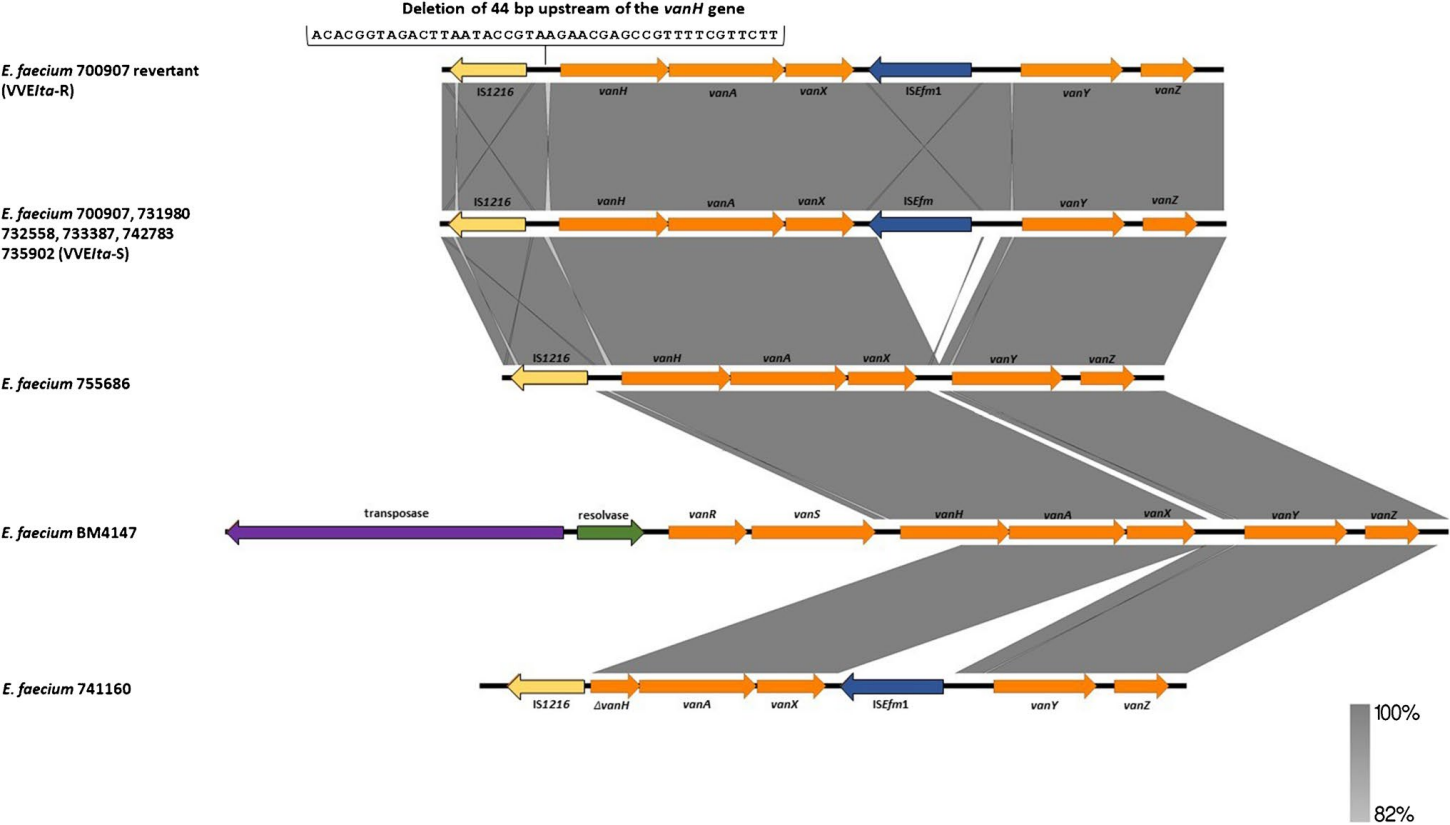
Linear map of the Tn*1546* prototype of *E. faecium* BM4147 (accession no. M97297.1) in comparison with defective Tn*1546* transposons of VVE*Ita*-S and VVE*Ita*-R isolates using Easyfig tool (https://mjsull.github.io/Easyfig/). The *van* gene cluster is shown in orange and the truncated *vanH* is indicated as *ΔvanH*. The positions and transcriptional direction of the ORFs are represented with arrows. Some antibiotic resistance determinants and relevant genes described in this study are shown

In order to investigate the genetic basis of vancomycin susceptibility of the 8 isolates, the nucleotide sequence of the Tn*1546* transposon has been thoroughly studied and compared to the prototype Tn*1546* (GenBank accession no. M97297). WGS analysis revealed that the transposase and resolvase genes were missing, and two types of *vanA* cluster have been found (Table 2 and Fig. 1): (i) the first type, found in 7 isolates, showed an intact *vanH/vanA/vanX* cluster, the lack of *vanR* gene and the deletion of *vanS* gene and (ii) the second type, found in the 741,160 strain, was completely devoid of both *vanR* and *vanS* genes and showed a deletion of the *vanH* gene.

The first type of *vanA* cluster showed the insertion of the IS*1216* element at the 3′ end of the *vanS* gene. This insertion caused the deletion of a 5789 bp region of the Tn*1546* wildtype including the *vanR* gene, a large region of *vanS* (only 14 bp at the 3′ end was maintained), the resolvase and the Tn*1546* transposase. It is well-known that IS elements are highly mobile and can cause Tn*1546* structural alterations [35].

vanR and vanS are part of a two-component signal transduction system that controls the expression of the *vanH*/*vanA*/*vanX* cluster mediating vancomycin resistance. Several papers reported that the total absence or modification of this system could be responsible for a *vanA* genotype/vancomycin-susceptible phenotype, as the transcription of the genes required for resistance cannot be regulated [6, 11, 13]. Furthermore, six out of seven isolates had an IS*Efm1* transposase between *vanX* and *vanY* genes.

The second type of *vanA* cluster found in the 741,160 strain had no *vanR* and *vanS* genes and also showed a deletion of 544 bp at the 5′-end of the *vanH* gene (only 425/969 bp of the *vanH* gene has been maintained) due to the insertion of an IS*1216* element. This IS*1216* element was present in the *vanA*-plasmid of the 741,160 strain in multiple copies and their transposition could have contributed to the deletion of the *vanH*. Moreover, also an IS*Efm1* element between *vanX* and *vanY* genes was detected.

It is well-known that a complete *vanH*/*vanA*/*vanX* operon is required for the development of a vancomycin-resistant phenotype [6]; thus, the presence of a truncated *vanH* gene could contribute to vancomycin susceptibility. For this reason, the 741,160 strain was not considered a VVE*Ita*-S.

### VVE*Ita*-S resistance phenotype reversion and its stability

We selected the VVE*Ita*-S 700907 isolate (the representative of the 7 strains with the type 1 *vanA* cluster) to verify its ability to revert to the vancomycin-resistant phenotype under laboratory conditions. We obtained adaptive mutants by plating the strain in agar supplemented with increasing concentrations of vancomycin. Mutants were able to evolve to full vancomycin resistance after a week, showing a vancomycin MIC of > 128 mg/L.

The 700,907 mutant (VVE*Ita*-R) was subjected to WGS and compared to the parental VVE*Ita*-S strain by BLASTN analysis. The mutant genome disclosed a 44 bp deletion (ACACGGTAGACTTAATACCGTAAGAACGAGCCGTTTTCGTTCTT) in the promoter region of *vanH*/*vanA*/*vanX* cluster causing its constitutive expression, as previously described (Fig. 1) [6, 11]. The VVE*Ita*-R strain showed unvaried high levels of resistance to vancomycin even after induction, confirming the constitutive expression of the *vanH/vanA/vanX* cluster. Wagner et al. suggested that an alternative promoter conveys *vanH*/*vanA*/*vanX* expression independently of the *vanR* activator [11]. Indeed, upstream of the *vanH* gene, we found the same consensus sequences of P2 and P3 promoters previously described by Wagner et al. [11]. Genome comparisons revealed no other relevant alterations that could be associated to the phenotypic differences between these isogenic strains.

We further investigated the stability of the vancomycin resistance phenotype of the VVE*Ita*-R under non-selective conditions (on antibiotic-free) for 15 days. After 5 days of passages, the revertant isolates were still resistant (MIC, > 128 mg/L); however, the resistance phenotype was unstable; since at the 11th day of growth in agar without vancomycin, all strains showed a reduced level of resistance (MIC, 8 mg/L) despite the *vanA* gene was still detected. These findings are consistent with the data of Wagner et al. which highlighted a temporal progression of most VVE-R isolates towards vancomycin susceptibility [6] in the absence of exposure to vancomycin.

In order to determine the genetic basis associated with the reduction of vancomycin resistance, a selected mutant strain (named VVE*Ita*-R1) was subjected to WGS. Comparing the region upstream of the *vanH* gene of the VVE*Ita*-R mutant highly resistant to vancomycin (MIC, > 128 mg/L) with that of the VVE*Ita*-R1 mutant (MIC, 8 mg/L), we found only one point mutation (C to A) located at the beginning of the truncated *vanS* gene.

### Plasmid location of *vanA* cluster

WGS analysis of the seven VVE*Ita*-S and the *E. faecium* 741,160 revealed that the *vanA* cluster was always localized on plasmids of size range 42–56 kb (G + C content 35%), all belonging to RepA_N replicon type (Table 2). Plasmids did not carry any antibiotic-resistance genes other than the *vanA* gene cluster. The relevant ORFs of the eight *vanA* plasmids are indicated in tables S2 to S9.

BLASTN analysis revealed that the 8 *vanA* plasmids were 99.36%–99.97% (coverage range 91–100%) identical to each other and overall showed the best coverage and nucleotide identity (33–37% and 98–99%, respectively) with the 83.6 kb plasmid (accession no. CP092571.1) of the *Enterococcus faecium* VRE-WC072. The high nucleotide identity of these plasmids harbored by the 8 different strains isolated from different wards suggested an intra-species spread of the same *vanA* plasmid among the hospital circulating enterococci.

**Fig. 2:**
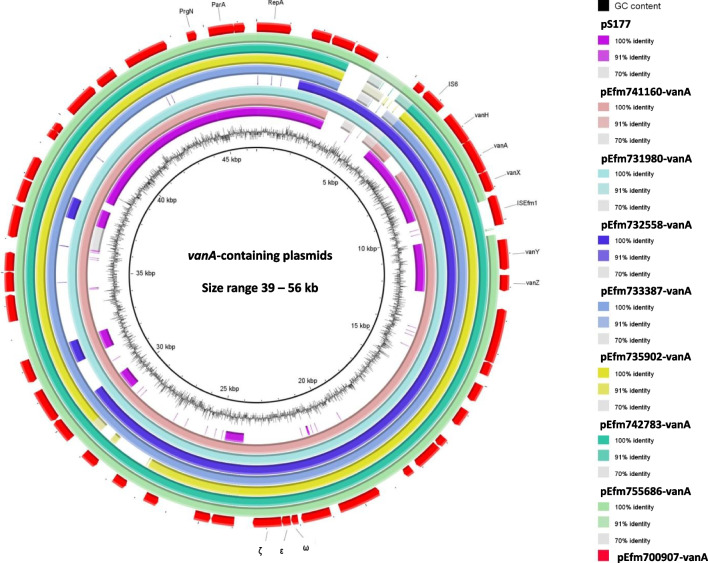
Circular maps of the *vanA*-containing plasmids of the 8 sequenced VVE*Ita*-S in comparison with pS177 plasmid using BRIG software. Plasmids and transposons included in the analysis were as follows: (inner to outer circles) pS177 of *E. faecium* S177 (accession no. HQ115078), pEfm741160-vanA of *E. faecium* 741,160 (accession no. OR234015), pEfm731980-vanA of *E. faecium* 731,980 (accession no. OR234011), pEfm732558-vanA of *E. faecium* 732,558 (accession no. OR251469), pEfm733387-vanA of *E. faecium* 733,387 (accession no. OR234012), pEfm735902-vanA of *E. faecium* 735,902 (accession no. OR298096), pEfm742783-vanA of *E. faecium* 742,783 (accession no. OR251470), pEfm755686-vanA of *E. faecium* 755,686 (accession no. OR262468), and pEfm700907-vanA of *E. faecium* 700,907 (accession no. OR208591). Red arrows indicate the position and orientation of the genes of the pEfm700907-vanA used as reference; some antibiotic resistance determinants and relevant genes described in this study are shown

Moreover, all eight *vanA* plasmids showed a DNA identity of 100% (coverage 48%) with the 39 kb pS177 plasmid (GenBank accession no. NC_014959) of the *E. faecium* strain from the USA, which typically carried this defective *vanA* gene cluster (Fig. 2).

In VVE isolates, the *vanA* gene cluster has been described also on a conjugative plasmid [9, 10, 14]. Despite several attempts, our strains were unable to transfer the *vanA* plasmid to the *E. faecium* 64/3 recipient at frequencies detectable under laboratory conditions. However, the high identity of plasmids carried by the eight different strains demonstrates that the dissemination of these *vanA* plasmids could occur by co-resident plasmids mediating the transfer.

The copy number of *vanA* plasmid was measured in the VVE*Ita*-S 700907 strain and in its relative mutants 700,907 VVE*Ita*-R and VVE*Ita*-R1. Both mutants harbored a higher copy number of the *vanA* plasmid compared with the VVE*Ita*-S 700907 parental strain, respectively, with a 17 ± onefold increase in mutant 700,907 VVE*Ita*-R1 and 40 ± onefold in mutant 700,907 VVE*Ita*-R consistently to their MIC values as previously reported by Wagner et al. [11].

## Conclusion

Here, we report the first identification of VVE*Ita*-S clinical *E. faecium* isolates in Italy resulting from deletions in the Tn*1546*. To the best of our knowledge, we report the first identification of VVE-S isolates ST1478 *pstS*-null.

We found that VVE-S with deletions only in the two-component signal transduction system vanR/vanS, exposed to increasing concentrations of vancomycin, could revert in vancomycin-resistant strains, as previously reported elsewhere. As revertants revealed the same 44-bp deletion in the *vanH*/*vanA*/*vanX* promoter region also found in other VVE-S strains (i.e., VVE*Swe*-R and VVE*Aus*-R), we hypothesized that the mechanism by which our VVE strains revert to resistant phenotype could be the use of an alternative and constitutive, *vanR*-independent promoter as reported by Wagner et al. [6, 11]. Moreover, the detection of a higher plasmid copy number in the revertant strains seems to be related to the reversion to a resistant phenotype, in line with previous studies [6, 11].

Since the identification of VRE routinely depends on phenotypic characterization and not on genotypic analysis, VVE isolates go unnoticed. It should be suggested to test enterococci by both genotypic and phenotypic methods [9] since vancomycin resistance might arise following the use of vancomycin in clinical settings for the therapy of VVE infections with the risk of treatment failures and severe impacts on public health.

### Supplementary Information

Below is the link to the electronic supplementary material.

Supplementary file1 (DOCX 23 KB)

Supplementary file2 (DOCX 21 KB)

Supplementary file3 (DOCX 23 KB)

Supplementary file4 (DOCX 24 KB)

Supplementary file5 (PDF 144 KB)

Supplementary file6 (PDF 137 KB)

Supplementary file7 (PDF 90 KB)

Supplementary file8 (DOCX 21 KB)

Supplementary file9 (DOCX 23 KB)

Supplementary file10 (DOCX 20 KB)

Supplementary file11 (DOCX 23 KB)

## Data Availability

The authors confirm that the data supporting the findings of this study are available within the article.
