# Supplementary material for: Genetic analysis of vancomycin-variable Enterococcus faecium clinical isolates in Italy
Source: Eur J Clin Microbiol Infect Dis. 2024 Jan 31;43(4):673–82. doi: 10.1007/s10096-024-04768-0 (PMC10965585; doi:10.1007/s10096-024-04768-0)
Supplement: Supplementary file 1 — Supplementary file1 (DOCX 23 KB) [file 10096_2024_4768_MOESM1_ESM.docx]

**Table S6.** Amino acid sequence identities/similarities of putative proteins encoded by the pEfm735902-vanA (GenBank accession no. OR298096).

---------------------------------------------------------------------------------------------------------------------------------------------------------------------------------------------------------------------------------------------------- BLASTP analysis*a* Size ----------------------------------------------------------------------------------------------------------------------------------------------------------------------------------------------------

ORF Start Stop (amino Predicted function % Amino acid

(bp) (bp) acids) Most significant database match Accession no. identity (% amino

acid similarity)

----------------------------------------------------------------------------------------------------------------------------------------------------------------------------------------------------------------------------------------------------

*orf1* 1 1041 346 Replication initiation protein Replication protein RepA [*Enterococcus faecium*] BDP48539.1 100 (100)

*orf2* 2343 1657 228 IS6 family transposase IS*6*-like element IS*1216* family transposase, partial [*E. faecium*] KAB7578479.1 99 (100)

*orf3* 3159 2473 228 IS6 family transposase IS6 family transposase [*Enterococcus faecalis*] TQB04140.1 99 (100)

*orf4* 3456 4424 322 D-lactate dehydrogenase VanH Vancomycin resistance protein VanH [*E. faecium*] ADO66796.1 100 (100)

*orf5* 4417 5448 343 D-alanine--(R)-lactate ligase D-alanine--(R)-lactate ligase VanA [*E. faecium*] HBM8952485.1 99 (100)

*orf6* 5454 6062 202 D-alanyl-D-alanine dipeptidase Vancomycin B-type resistance protein VanX [*E. faecium*] HAQ5904304.1 100 (100)

∆*orf7* 7030 6182 282 IS*982* family transposase IS982-like element ISEfm1 family transposase [*Enterococcus faecium*] WP_127824070.1 99 (100)

*orf8* 7527 8438 326 D-Ala-D-Ala dipeptidase/carboxypeptidase D-Ala-D-Ala carboxypeptidase [*Enterococcus faecium TX0133a01*] EFR67896.1 100 (100)

*orf9* 8591 9076 161 Teicoplanin resistance protein VanZ Glycopeptide resistance protein VanZ [*Enterococcus faecium*] HDL1085041.1 99 (100)

*orf10* 9442 9846 134 IS200/IS605 family transposase IS200/IS605-like element ISEfa4 family transposase [*Bacteria*] WP_002287522.1 100 (100)

*orf11* 9863 11011 382 IS200/IS605 family element IS200/IS605 family element RNA-guided endonuclease TnpB [*Bacteria*] WP_002287525.1 100 (100)

*orf12* 13132 11492 546 Mercuric ion reductase Mercury(II) reductase [*Enterococcus faecium*] HCD4411652.1 100 (100)

*orf13* 13544 13146 132 Mercuric resistance regulatory protein, MerR MerR family transcriptional regulator [*E. faecium* Com15] EEV63162.1 100 (100)

*orf14* 13861 14412 183 Tyrosine recombinase Phage integrase [*E. faecium* ATCC 8459] AGE31333.1 100 (100)

*orf15* 14725 15267 180 Hypothetical protein T641_10295 [*E. faecium* MRSN 4777] KKJ72108.1 100 (100)

*orf16* 15778 16068 96 IS*3* family transposase Transposase [*E. faecium*] ALZ53562.1 100 (100)

*orf17* 16104 16940 278 IS*3* family transposase IS*3* family transposase [*E. faecium*] WP_154213969.1 100 (100)

*orf18* 17134 17400 88 YfhO family protein [*Enterococcus faecium*] MBH0800404.1 99 (100)

*orf19* 17482 17778 98 Hypothetical protein [*Enterococcus*] WP_002307630.1 100 (100)

*orf20* 17788 17994 68 Hypothetical protein [*Enterococcus*] WP_002295288.1 100 (100)

*orf21* 19597 18302 431 ISEfa5 family transposase ISL3-like element ISEfa5 family transposase [*Enterococcus faecium*] WP_199004470.1 99 (100)

*orf22* 19890 20786 284 ParA family protein [*Enterococcus sp.*] NLM66716.1 100 (100)

*orf23* 20884 21093 69 Transcriptional regulator Omega protein [*Enterococcus faecium*] MBK4807767.1 99 (98)

*orf24* 21111 21383 90 Epsilon antitoxin Antitoxin [*Enterococcus faecium*] WP_104770826.1 99 (100)

*orf25* 21385 21894 287 Zeta toxin Zeta toxin family protein [*Enterococcus faecium*] WP_113827883.1 99 (99)

*orf26* 22614 21928 228 IS6 family transposase IS6-like element IS1216 family transposase [*Enterococcus faecium*] MCZ2247035.1 99 (99)

*∆orf27* 23551 23171 126 Zeta toxin Zeta-toxin [Enterococcus faecium] TYQ34725.1 100 (100)

*orf28* 23622 24308 228 IS6 family transposase IS6-like element IS1216 family transposase [*Enterococcus faecium*] MCZ2247035.1 99 (99)

*orf29* 24819 24331 162 Replication protein Rep Replication protein Rep [*Enterococcus faecium*] AWB15732.1 97 (99)

*orf30* 26194 26598 134 IS200/IS605 family transposase IS200/IS605-like element ISEfa4 family transposase [*Bacteria*] WP_002287522.1 100 (100)

*orf31* 26615 27763 382 IS200/IS605 family element IS200/IS605 family element RNA-guided endonuclease TnpB [*Bacteria*] WP_002287525.1 100 (100)

*orf32* 28274 27900 124 Hypothetical protein [*Enterococcus faecium*] WP_060799182.1 100 (100)

*orf33* 29445 28912 177 DUF536 domain-containing protein [Bacteria] WP_002363576.1 100 (100)

*orf34* 30410 29466 314 Plasmid replication initiation protein Replication initiation protein [*Enterococcus faecium*] WP_224471684.1 99 (100)

*orf35* 31943 31254 229 Hypothetical protein [*Enterococcus faecium*] HAQ5061312.1 99 (100)

*orf36* 33102 32188 304 Mobilization protein Relaxase/mobilization nuclease domain-containing protein [*Bacteria*] WP_010782659.1 100 (100)

*orf37* 33464 33084 126 Mobilization protein MobC family plasmid mobilization relaxosome protein [*Bacteria*] WP_010782660.1 100 (100)

*orf38* 34220 34906 228 IS6 family transposase IS6-like element IS1216 family transposase [*Enterococcus faecium*] MCZ2247035.1 99 (99)

*∆orf39* 35213 36886 557 Hypothetical protein [*Enterococcus faecium*] WP_002326343.1 100 (100)

*orf40* 37350 38159 269 IS30 family transposase IS30 family transposase [Enterococcus faecalis] ARQ19074.1 100 (100)

*orf41* 38246 38851 201 Fic domain protein Fic family protein [*Enterococcus faecium*] WP_139910168.1 99 (100)

*orf42* 38867 39439 109 Site-specific recombinase Recombinase family protein [*Enterococcus faecium*] HAQ4760375.1 99 (99)

*orf43* 40831 39872 319 Integrase, catalytic region IS30-like element IS1252 family transposase [*Enterococcus faecium*] MBJ1016605.1 99 (100)

*orf44* 41645 40959 228 IS6 family transposase IS6-like element IS1216 family transposase [*Enterococcus faecium*] MCZ2247035.1 99 (99)

*orf45* 41701 42396 231 Hypothetical protein [*Enterococcus*] WP_002326819.1 100 (100)

*orf46* 42745 42443 100 Hypothetical protein [*Enterococcus faecium*] AAO52834.1 100 (100)

*orf47* 43087 43356 89 YefM protein Toxin-antitoxin system Phd/YefM family antitoxin [*Enterococcus faecium*] EGP5080672.1 99 (98)

*orf48* 43349 43606 85 YoeB toxin protein Txe/YoeB family addiction module toxin [*Enterococcus faecium*] MBK4852254.1 100 (100)

*orf49* 44065 45069 334 Hypothetical protein, partial [*Enterococcus faecium*] WP_230853401.1 100 (100)

*orf50* 45848 45234 204 Site-specific recombinase Recombinase family protein [*Bacteria*] WP_001261742.1 100 (100)

*orf51* 46298 47623 441 ImpB/MucB/SamB family protein Y-family DNA polymerase [*Enterococcus faecium*] HAQ7475362.1 99 (100)

*orf52* 47589 47966 116 DNA-directed RNA polymerase beta subunit Hypothetical protein [*Enterococcus faecium*] HBL3392154.1 99 (99)

*orf53* 48278 48568 96 Replication control protein PrgN Type III secretion system protein PrgN [*Enterococcus faecium*] HBD0771398.1 99 (100)

*orf54* 48936 49724 262 Partitioning protein ParA ParA family protein [*Enterococcus faecium*] HAP6146794.1 99 (99)

*orf55* 49711 50037 109 Hypothetical protein, partial [*Enterococcus faecium*] WP_154494709.1 99 (100)

----------------------------------------------------------------------------------------------------------------------------------------------------------------------------------------------------------------------------------------------------

*^a^*For each ORF, only the most significant identity detected is listed
