# Supplementary material for: Genetic analysis of vancomycin-variable Enterococcus faecium clinical isolates in Italy
Source: Eur J Clin Microbiol Infect Dis. 2024 Jan 31;43(4):673–82. doi: 10.1007/s10096-024-04768-0 (PMC10965585; doi:10.1007/s10096-024-04768-0)
Supplement: Supplementary file 2 — Supplementary file2 (DOCX 21 KB) [file 10096_2024_4768_MOESM2_ESM.docx]

**Table S7** Amino acid sequence identities/similarities of putative proteins encoded by the pEfm741160-vanA (GenBank accession no. OR234015) of the *E. faecium* 741160.

---------------------------------------------------------------------------------------------------------------------------------------------------------------------------------------------------------------------------------------------------- BLASTP analysis*a* Size ----------------------------------------------------------------------------------------------------------------------------------------------------------------------------------------------------

ORF Start Stop (amino Predicted function % Amino acid

(bp) (bp) acids) Most significant database match Accession no. identity (% amino

acid similarity)

----------------------------------------------------------------------------------------------------------------------------------------------------------------------------------------------------------------------------------------------------

*orf1* 1 1041 346 Replication initiation protein Replication protein RepA [*Enterococcus faecium*] BDP48539.1 100 (100)

*orf2* 2463 1777 228 IS*6* family transposase IS*6*-like element IS*1216* family transposase [*Enterococcus* *faecalis*] PQE58815.1 99 (100)

*Δorf3* 2519 2956 145 D-lactate dehydrogenase VanH D-lactate dehydrogenase VanH [*Enterococcus* *faecium*] WP_144319791.1 99 (97)

*orf4* 2949 3980 343 D-alanine--(R)-lactate ligase D-alanine--(R)-lactate ligase VanA [*Enterococcus* *faecium*] WP_063856521.1 99 (100)

*orf5* 3986 4594 202 D-alanyl-D-alanine dipeptidase D-Ala-D-Ala dipeptidase VanX-A [*Bacteria*] WP_000402347.1 100 (100)

*orf6* 5622 4714 302 IS*982* family transposase IS*982*-like element ISEfm1 family transposase [*Bacteria*] WP_002295743.1 100 (100)

*orf7* 6070 6981 303 D-Ala-D-Ala dipeptidase/carboxypeptidase D-Ala-D-Ala carboxypeptidase VanY-A [*Bacteria*] WP_001812592.1 100 (100)

*orf8* 7134 7619 161 Teicoplanin resistance protein VanZ Glycopeptide resistance protein VanZ-A [*Bacteria*] WP_000516404.1 100 (100)

*orf9* 9784 8144 546 Mercuric ion reductase Mercury (II) reductase [*Enterococcus* *faecium*] EGP5032460.1 99 (100)

*orf10* 10196 9798 132 Mercuric resistance regulatory protein, MerR MerR family transcriptional regulator [*Bacteria*] WP_002301360.1 100 (100)

*orf11* 10513 11064 183 Prophage λSa2, site-specific recombinase Tyrosine-type recombinase/integrase [*Enterococcaceae*] WP_002307628.1 100 (100)

*orf12* 11377 11919 180 Hypothetical protein [*E. faecium*] MBK4849403.1 99 (100)

*orf13* 12430 12720 96 IS*3* family transposase Transposase [*E. faecium*] ALZ53562.1 100 (100)

*orf14* 12756 13592 278 IS*3* family transposase IS*3* family transposase [*E. faecium*] WP_154213969.1 100 (100)

*orf15* 13786 14052 88 YfhO family protein [*Enterococcus faecium*] MBH0800404.1 99 (100)

*orf16* 16249 14954 431 ISEfa5 family transposase ISL3-like element ISEfa5 family transposase [*Enterococcus faecium*] WP_151076461.1 99 (100)

*Δorf17* 16584 17438 284 ParA family protein [*Enterococcus* *faecalis*] WP_089202011.1 100 (100)

*orf18* 17536 17745 69 Transcriptional regulator Omega protein [*Enterococcus faecium*] MBK4807767.1 99 (98)

*orf19* 17763 18035 90 Epsilon antitoxin Antitoxin [*Enterococcus faecium*] WP_104770826.1 99 (100)

*orf20* 18037 18900 287 Zeta toxin Zeta toxin family protein [*Enterococcus faecium*] WP_113827883.1 99 (99)

*orf21* 19457 20143 228 IS*6* family transposase IS6-like element IS1216 family transposase [*Enterococcus faecium*] MCZ2247035.1 99 (99)

*Δorf22* 20654 20166 162 Plasmid replication initiation protein Replication initiation protein [*Enterococcus* *faecium*] WP_159067278.1 94 (96)

*Δorf23* 21510 21250 86 Mobilization protein MobC family plasmid mobilization relaxosome protein [*E*. *faecium*] WP_196003520.1 94 (100)

*orf24* 22228 21803 141 Hypothetical protein [*Enterococcus faecium*] EGP5549539.1 99 (99)

*orf25* 23302 22886 138 Hypothetical protein [*Enterococcus faecium*] WP_195424410.1 99 (100)

*orf26* 24458 23961 165 DUF536 domain-containing protein [*Enterococcus faecium*] WP_002347002.1 95 (97)

*orf27* 25065 25751 228 IS6 family transposase IS6-like element IS1216 family transposase [*Enterococcus faecium*] MCZ2247035.1 99 (99)

*orf28* 26795 25785 336 Hypothetical protein, partial [*Enterococcus faecium*] MCZ1334167.1 100 (100)

*orf29* 27788 27102 228 IS6 family transposase IS6-like element IS1216 family transposase [*Enterococcus faecium*] MCZ2247035.1 99 (99)

*orf30* 27844 28548 234 Hypothetical protein [*Enterococcus* *faecium*] MCZ1768805.1 100 (100)

*Δorf31* 29012 29821 269 Integrase, catalytic region IS30 family transposase [*Enterococcus*] WP_228012590.1 99 (100)

*orf32* 29908 30513 201 Fic domain protein Fic family protein [*Enterococcus* *faecium*] WP_139910168.1 99 (100)

*orf33* 30529 31101 190 Site-specific recombinase recombinase family protein [*Bacteria*] WP_000170424.1 100 (100)

*orf34* 32493 31534 319 Integrase, catalytic region IS30-like element IS1252 family transposase [Enterococcus faecium] MBJ1016605.1 99 (100)

*orf35* 33307 32621 228 IS6 family transposase IS6-like element IS1216 family transposase [*Enterococcus faecium*] MCZ2247035.1 99 (99)

*orf36* 33363 34058 231 Hypothetical protein [*Enterococcus*] WP_002326819.1 100 (100)

*orf37* 34749 35018 89 YefM protein Toxin-antitoxin system Phd/YefM family antitoxin [*Enterococcus faecium*] EGP5080672.1 99 (98)

*orf38* 35011 35268 85 YoeB toxin protein Txe/YoeB family addiction module toxin [*Enterococcus faecium*] MBK4852254.1 100 (100)

*orf39* 35727 36731 334 Hypothetical protein, partial [*Enterococcus* *faecium*] WP_230853401.1 100 (100)

*orf40* 37510 36896 204 Site-specific recombinase Recombinase family protein [*Bacteria*] WP_001261742.1 100 (100)

*orf41* 37960 39285 441 ImpB/MucB/SamB family protein Y-family DNA polymerase [*Enterococcus faecium*] HAQ7475362.1 99 (100)

*orf42* 39940 40230 96 Replication control protein PrgN Type III secretion system protein PrgN [*Enterococcus faecium*] HBD0771398.1 99 (100)

*orf43* 40598 41386 262 Partitioning protein ParA ParA family protein [*Enterococcus faecium*] HAP6146794.1 99 (99)

*orf44* 41373 41699 108 Hypothetical protein [*Enterococcus* *faecium*] WP_154494709.1 99 (100)

----------------------------------------------------------------------------------------------------------------------------------------------------------------------------------------------------------------------------------------------------

*^a^*For each ORF, only the most significant identity detected is listed
