# Supplementary material for: Genetic analysis of vancomycin-variable Enterococcus faecium clinical isolates in Italy
Source: Eur J Clin Microbiol Infect Dis. 2024 Jan 31;43(4):673–82. doi: 10.1007/s10096-024-04768-0 (PMC10965585; doi:10.1007/s10096-024-04768-0)
Supplement: Supplementary file 3 — Supplementary file3 (DOCX 23 KB) [file 10096_2024_4768_MOESM3_ESM.docx]

**Table S8.** Amino acid sequence identities/similarities of putative proteins encoded by the pEfm742783-vanA (GenBank accession no. OR251470) of the *E. faecium* 742783.

---------------------------------------------------------------------------------------------------------------------------------------------------------------------------------------------------------------------------------------------------- BLASTP analysis*a* Size ----------------------------------------------------------------------------------------------------------------------------------------------------------------------------------------------------

ORF Start Stop (amino Predicted function % Amino acid

(bp) (bp) acids) Most significant database match Accession no. identity (% amino

acid similarity)

----------------------------------------------------------------------------------------------------------------------------------------------------------------------------------------------------------------------------------------------------

*orf1* 1 1041 346 Replication initiation protein A Replication protein RepA [*Enterococcus faecium*] BDP48539.1 100 (100)

Δ*orf2* 2073 1657 138 IS*6* family transposase IS*6*-like element IS*1216* family transposase, partial [*Enterococcus faecium*] WP_080489831.1 99 (100)

*orf3* 2853 2167 228 IS*6* family transposase IS*6* family transposase [*Enterococcus faecalis*] ARQ19076.1 99 (100)

*orf4* 3611 2901 236 Plasmid replication protein Replication initiation protein [Enterococcus faecium] MCZ1195426.1 100 (100)

*orf5* 5039 5323 94 Hypothetical protein [Enterococcus faecium] WP_151497033.1 99 (100)

*orf6* 6077 5391 228 IS*6* family transposase IS*6*-like element IS*1216* family transposase [*Enterococcus* *faecium*] MCZ2247035.1 99 (100)

*orf7* 6374 7342 322 D-lactate dehydrogenase VanH VanH-A/VanH-Pt family D-lactate dehydrogenase [*Enterococcus* *faecium*] WP_198832401.1 99 (100)

*orf8* 7335 8366 343 D-alanine--(R)-lactate ligase D-alanine--(R)-lactate ligase VanA [*E. faecium*] HBM8952485.1 99 (100)

*orf9* 8372 8980 202 D-alanyl-D-alanine dipeptidase D-Ala-D-Ala dipeptidase VanX [*Enterococcus* *faecalis*] WP_151069977.1 99 (100)

*orf10* 10008 9100 302 IS*982* family transposase IS*982*-like element ISEfm1 family transposase [*Enterococcus* *faecium*] WP_198791813.1 99 (100)

*orf11* 10456 11367 303 D-Ala-D-Ala dipeptidase/carboxypeptidase VanY D-Ala-D-Ala carboxypeptidase [*Enterococcus* *faecium*] WP_139913473.1 99 (100)

*orf12* 11520 12005 161 Teicoplanin resistance protein VanZ Glycopeptide resistance protein VanZ-A [*Enterococcus* *faecium*] HDL1085041.1 99 (100)

*orf13* 14170 12530 546 Mercuric ion reductase Mercury (II) reductase [*Enterococcus* *faecium*] EGP5032460.1 99 (100)

*orf14* 14582 14184 132 Mercuric resistance regulatory protein, MerR MerR family transcriptional regulator [*Bacteria*] WP_002301360.1 100 (100)

*orf15* 14899 15450 183 Prophage λSa2, site-specific recombinase Tyrosine-type recombinase/integrase [*Enterococcaceae*] WP_002307628.1 100 (100)

*orf16* 15763 16305 180 Hypothetical protein [*E. faecium*] MBK4849403.1 99 (100)

*orf17* 16816 17106 96 IS*3* family transposase Transposase [*E. faecium*] ALZ53562.1 100 (100)

*orf18* 17142 17978 278 IS*3* family transposase IS*3* family transposase [*E. faecium*] WP_154213969.1 100 (100)

*orf19* 18172 18438 88 YfhO family protein [*Enterococcus faecium*] MBH0800404.1 99 (100)

*orf20* 20635 19340 431 IS*Efa5* family transposase IS*L3*-like element IS*Efa5* family transposase [*Enterococcus* *faecium*] WP_151076461.1 99 (100)

*orf21* 20928 21824 298 ParA family protein [*Enterococcus* *faecalis*] WP_089202011.1 100 (100)

*orf22* 21922 22131 69 Transcriptional regulator Omega protein [*Enterococcus faecium*] MBK4807767.1 99 (98)

*orf23* 22149 22421 90 Epsilon antitoxin Antitoxin [*Enterococcus faecium*] WP_104770826.1 99 (100)

*orf24* 22423 23286 287 Zeta toxin Zeta toxin family protein [*Enterococcus faecium*] WP_113827883.1 99 (99)

*orf25* 23843 24529 228 IS*6* family transposase IS*6*-like element IS*1216* family transposase [*Enterococcus faecium*] MCZ2247035.1 99 (99)

*orf26* 25040 24552 162 Plasmid replication initiation protein Replication protein Rep [*Enterococcus faecium*] AWB15732.1 100 (100)

*orf27* 26619 26194 141 Hypothetical protein [*Enterococcus faecium*] EGP5549539.1 99 (99)

*orf28* 27693 27277 138 Hypothetical protein [*Enterococcus faecium*] WP_195424410.1 99 (100)

*orf29* 28849 28352 165 DUF536 domain-containing protein [*Enterococcus faecium*] WP_002347002.1 100 (100)

*orf30* 29456 30142 228 IS*6* family transposase IS*6*-like element IS*1216* family transposase [*Enterococcus faecium*] MCZ2247035.1 99 (99)

*orf31* 31186 30176 336 Hypothetical protein [*Enterococcus* *faecium*] MCZ1334167.1 100 (100)

*orf32* 32179 31493 228 IS*6* family transposase IS*6*-like element IS*1216* family transposase [*Enterococcus faecium*] MCZ2247035.1 99 (99)

*orf33* 32235 32939 234 Hypothetical protein [*Enterococcus* *faecium*] MCZ1768805.1 100 (100)

*orf34* 33403 34212 269 Integrase, catalytic region IS30 family transposase [*Enterococcus* *faecalis*] ARQ19074.1 100 (100)

*orf35* 34299 34904 201 Fic domain protein Fic family protein [*Enterococcus* *faecium*] WP_139910168.1 99 (100)

*orf36* 34920 35492 190 Site-specific recombinase Recombinase family protein [*Enterococcus* *faecium*] WP_169038514.1 99 (99)

*orf37* 36884 35925 319 Integrase, catalytic region IS30-like element IS1252 family transposase [*Enterococcus faecium*] MBJ1016605.1 99 (100)

*orf38* 37698 37012 228 IS*6* family transposase IS*6*-like element IS*1216* family transposase [*Enterococcus faecium*] MCZ2247035.1 99 (100)

*orf39* 37754 38449 231 Hypothetical protein [*Enterococcus*] WP_002326819.1 100 (100)

*orf40* 39140 39409 89 YefM protein Toxin-antitoxin system Phd/YefM family antitoxin [*Enterococcus faecium*] EGP5080672.1 99 (98)

*orf41* 39402 39659 85 YoeB toxin protein Txe/YoeB family addiction module toxin [*Enterococcus faecium*] MBK4852254.1 100 (100)

*orf42* 40118 41122 334 Hypothetical protein [*Enterococcus* *faecium*] NTN88087.1 99 (100)

*orf43* 41901 41287 204 Site-specific recombinase Recombinase family protein [*Bacteria*] WP_001261742.1 100 (100) *orf44* 42354 43676 440 ImpB/MucB/SamB family protein Y-family DNA polymerase [*Enterococcus faecium*] HAQ7475362.1 99 (100)

*Δorf45* 44331 44621 96 Replication control protein PrgN type III secretion system protein PrgN [Enterococcus faecium] WP_084803251.1 100 (100)

*orf46* 44989 45777 262 Partitioning protein ParA ParA family protein [*Enterococcus faecium*] HAP6146794.1 99 (99)

*orf47* 45761 46090 109 Hypothetical protein, partial [*Enterococcus faecium*] WP_154494709.1 99 (100)

----------------------------------------------------------------------------------------------------------------------------------------------------------------------------------------------------------------------------------------------------

*^a^*For each ORF, only the most significant identity detected is listed
