# Supplementary material for: Genetic analysis of vancomycin-variable Enterococcus faecium clinical isolates in Italy
Source: Eur J Clin Microbiol Infect Dis. 2024 Jan 31;43(4):673–82. doi: 10.1007/s10096-024-04768-0 (PMC10965585; doi:10.1007/s10096-024-04768-0)
Supplement: Supplementary file 4 — Supplementary file4 (DOCX 24 KB) [file 10096_2024_4768_MOESM4_ESM.docx]

**Table S9.** Amino acid sequence identities/similarities of putative proteins encoded by the pEfm755686-vanA (GenBank accession no. OR262468).

---------------------------------------------------------------------------------------------------------------------------------------------------------------------------------------------------------------------------------------------------- BLASTP analysis*a* Size ----------------------------------------------------------------------------------------------------------------------------------------------------------------------------------------------------

ORF Start Stop (amino Predicted function % Amino acid

(bp) (bp) acids) Most significant database match Accession no. identity (% amino

acid similarity)

----------------------------------------------------------------------------------------------------------------------------------------------------------------------------------------------------------------------------------------------------

*orf1* 1 1041 346 Replication initiation protein Replication protein RepA [*Enterococcus faecium*] BDP48539.1 100 (100)

*orf2* 2343 1657 228 IS6 family transposase IS*6*-like element IS*1216* family transposase, partial [*E. faecium*] KAB7578479.1 99 (100)

*orf3* 3159 2473 228 IS6 family transposase IS6 family transposase [*Enterococcus faecalis*] TQB04140.1 99 (100)

*orf4* 3311 4327 338 Replication protein Replication initiation factor [*Enterococcus faecium* *V689*] EJX43429.1 100 (100)

*orf5* 4330 4563 77 Hypothetical protein [*Bacilli*] WP_002298421.1 100 (100)

*orf6* 4556 4975 139 Hypothetical protein [*E. faecium*] HCA4994652.1 99 (100)

*orf7* 4984 5292 102 Hypothetical protein [*Bacteria*] WP_002298419.1 100 (100)

*orf8* 5296 5658 120 Hypothetical protein [*E. faecium*] HBL3257748.1 98 (99)

*∆orf9* 5815 7287 490 Mob mobilization protein [*Enterococcus faecium 1,231,410*] EEV55046.1 99 (99)

*orf10* 7686 8702 338 Replication protein Replication initiation factor [*Enterococcus faecium V689*] EJX43429.1 100 (100)

*orf11* 8705 8938 77 Hypothetical protein [*Bacilli*] WP_002298421.1 100 (100)

*orf12* 8931 9350 139 Hypothetical protein [*E. faecium*] HCA4994652.1 99 (100)

*orf13* 9359 9667 102 Hypothetical protein [*Bacteria*] WP_002298419.1 100 (100)

*orf14* 9671 10033 120 Hypothetical protein [*E. faecium*] HBL3257748.1 98 (99)

*∆orf15* 10190 11662 490 Mob mobilization protein [*Enterococcus faecium 1,231,410*] EEV55046.1 99 (99)

*orf16* 12726 12040 228 IS6 family transposase IS*6* family transposase [*E. faecium*] MCZ2247035.1 99 (99)

*orf17* 13023 13991 322 D-lactate dehydrogenase VanH Vancomycin resistance protein VanH [*E. faecium*] ADO66796.1 100 (100)

*orf18* 13984 15015 343 D-alanine--(R)-lactate ligase D-alanine--(R)-lactate ligase VanA [*E. faecium*] HBM8952485.1 99 (100)

*orf19* 15012 15629 202 D-alanyl-D-alanine dipeptidase Vancomycin B-type resistance protein VanX [*E. faecium*] HAQ5904304.1 100 (100)

*orf20* 15936 16916 326 D-Ala-D-Ala dipeptidase/carboxypeptidase D-Ala-D-Ala carboxypeptidase [*Enterococcus faecium TX0133a01*] EFR67896.1 100 (100)

*orf21* 17069 17554 161 Teicoplanin resistance protein VanZ Glycopeptide resistance protein VanZ [*Enterococcus faecium*] HDL1085041.1 99 (100)

*orf22* 19719 18079 546 Mercuric ion reductase Mercury(II) reductase [*Enterococcus faecium*] HCD4411652.1 100 (100)

*orf23* 20131 19733 132 Mercuric resistance regulatory protein, MerR MerR family transcriptional regulator [*E. faecium* Com15] EEV63162.1 100 (100)

*orf24* 20448 20999 183 Tyrosine recombinase Phage integrase [*E. faecium* ATCC 8459] AGE31333.1 100 (100)

*orf25* 21312 21854 180 Hypothetical protein T641_10295 [*E. faecium* MRSN 4777] KKJ72108.1 100 (100)

*orf26* 22365 22655 96 IS*3* family transposase Transposase [*E. faecium*] ALZ53562.1 100 (100)

*orf27* 22691 23527 278 IS*3* family transposase IS*3* family transposase [*E. faecium*] WP_154213969.1 100 (100)

*orf28* 23721 23987 88 YfhO family protein [*Enterococcus faecium*] MBH0800404.1 99 (100)

*orf29* 24069 24365 98 Hypothetical protein [*Enterococcus*] WP_002307630.1 100 (100)

*orf30* 24375 24581 68 Hypothetical protein [*Enterococcus*] WP_002295288.1 100 (100)

*orf31* 26184 24889 431 ISEfa5 family transposase ISL3-like element ISEfa5 family transposase [*Enterococcus faecium*] WP_199004470.1 99 (100)

*orf32* 26519 27373 284 ParA family protein [*Enterococcus sp.*] NLM66716.1 100 (100)

*orf33* 27471 27680 69 Transcriptional regulator Omega protein [*Enterococcus faecium*] MBK4807767.1 99 (98)

*orf34* 27698 27970 90 Epsilon antitoxin Antitoxin [*Enterococcus faecium*] WP_104770826.1 99 (100)

*orf35* 27972 28835 287 Zeta toxin Zeta toxin family protein [*Enterococcus faecium*] WP_113827883.1 99 (99)

*orf36* 29392 30078 228 IS6 family transposase IS6-like element IS1216 family transposase [*Enterococcus faecium*] MCZ2247035.1 99 (99)

*orf37* 31819 32103 94 Hypothetical protein [*Enterococcus faecium*] WP_266119626.1 99 (100)

*orf38* 32106 32522 138 Hypothetical protein [*Enterococcus faecium*] WP_208284707.1 99 (100)

*orf39* 33336 32659 162 Replication protein Rep Replication protein Rep [*Enterococcus faecium*] AWB15732.1 97 (99)

*orf40* 33415 34101 228 IS6 family transposase IS6-like element IS1216 family transposase [*Enterococcus faecium*] MCZ2247035.1 99 (99)

*orf41* 34612 34124 162 Plasmid replication initiation protein Replication protein Rep [*Enterococcus faecium*] AWB15732.1 97 (99))

*orf42* 36166 35741 141 Hypothetical protein [*Enterococcus faecium*] EGP5549539.1 99 (99)

*orf43* 37240 36824 138 Hypothetical protein [*Enterococcus faecium*] WP_195424410.1 99 (100)

*orf44* 37523 37242 93 Hypothetical protein [*Enterococcus*] WP_002296853.1 100 (100)

*orf45* 38396 37899 165 DUF536 domain-containing protein [*Enterococcus faecium*] WP_002347002.1 100 (100)

*orf46* 39003 39689 228 IS6 family transposase IS6-like element IS1216 family transposase [*Enterococcus faecium*] MCZ2247035.1 99 (99)

*orf47* 39996 41669 558 Hypothetical protein, partial [*Enterococcus*] WP_002326343.1 100 (100)

*orf48* 42133 42942 269 Integrase, catalytic region IS30 family transposase [*Enterococcus*] WP_228012590.1 99 (100)

*orf49* 43029 43634 201 Fic domain protein Fic family protein [*Enterococcus faecium*] WP_139910168.1 99 (100)

*orf50* 43650 44222 109 Site-specific recombinase Recombinase family protein [*Enterococcus faecium*] HAQ4760375.1 99 (99)

*orf51* 45819 44479 446 Transposase ISL3 family transposase [*Enterococcus*] WP_002303667.1 100 (100)

*orf52* 47156 46197 319 Integrase, catalytic region IS30-like element IS1252 family transposase [*Enterococcus faecium*] MBJ1016605.1 99 (100)

*orf53* 47970 47284 228 IS6 family transposase IS6-like element IS1216 family transposase [*Enterococcus faecium*] MCZ2247035.1 99 (99)

*orf54* 48026 48721 231 Hypothetical protein [*Enterococcus*] WP_002326819.1 100 (100)

*orf55* 49070 48768 100 Hypothetical protein [*Enterococcus faecium*] AAO52834.1 100 (100)

*orf56* 49412 49681 89 YefM protein Toxin-antitoxin system Phd/YefM family antitoxin [*Enterococcus faecium*] EGP5080672.1 99 (98)

*orf57* 49674 49931 85 YoeB toxin protein Txe/YoeB family addiction module toxin [*Enterococcus faecium*] MBK4852254.1 100 (100)

*orf58* 50390 51394 334 Hypothetical protein, partial [*Enterococcus faecium*] WP_230853401.1 100 (100)

*orf59* 52173 51559 204 Site-specific recombinase Recombinase family protein [*Bacteria*] WP_001261742.1 100 (100)

*orf60* 52623 53948 441 ImpB/MucB/SamB family protein Y-family DNA polymerase [*Enterococcus faecium*] HAQ7475362.1 99 (100)

*orf61* 53941 54291 116 DNA-directed RNA polymerase beta subunit Hypothetical protein [*Enterococcus faecium*] HBL3392154.1 99 (99)

*orf62* 54603 54893 96 Replication control protein PrgN Type III secretion system protein PrgN [*Enterococcus faecium*] HBD0771398.1 99 (100)

*orf63* 55261 56049 262 Partitioning protein ParA ParA family protein [*Enterococcus faecium*] HAP6146794.1 99 (99)

*orf64* 56036 56362 109 Hypothetical protein, partial [*Enterococcus faecium*] WP_154494709.1 99 (100)

----------------------------------------------------------------------------------------------------------------------------------------------------------------------------------------------------------------------------------------------------

*^a^*For each ORF, only the most significant identity detected is listed
