# Supplementary material for: Genetic analysis of vancomycin-variable Enterococcus faecium clinical isolates in Italy
Source: Eur J Clin Microbiol Infect Dis. 2024 Jan 31;43(4):673–82. doi: 10.1007/s10096-024-04768-0 (PMC10965585; doi:10.1007/s10096-024-04768-0)

Figure S1. SmaI-PFGE profiles of the 8 selected *E. faecium* VVE-S strains. Lane 1: *E. faecium* 700907 (A pulsotype); Lane 2: *E. faecium* 741160 (A1 pulsotype); Lane 3: *E. faecium* 731980 (A2 pulsotype); Lane 4: *E. faecium* 755686 (C pulsotype); Lane 5: *E. faecium* 742783 (D pulsotype); Lane 6: *E. faecium* 735902 (E pulsotype); Lane 7: *E. faecium* 732558 (B pulsotype); Lane 8: *E. faecium* 733387 (B1 pulsotype). According to the criteria of Tenover [29] closely related strains (restriction patterns showing two to three different bands) were assigned to the same pulsotype (e.g. A, A1, A2) whereas unrelated strains, with profiles differing in 7 or more bands, were included in different pulsotypes.

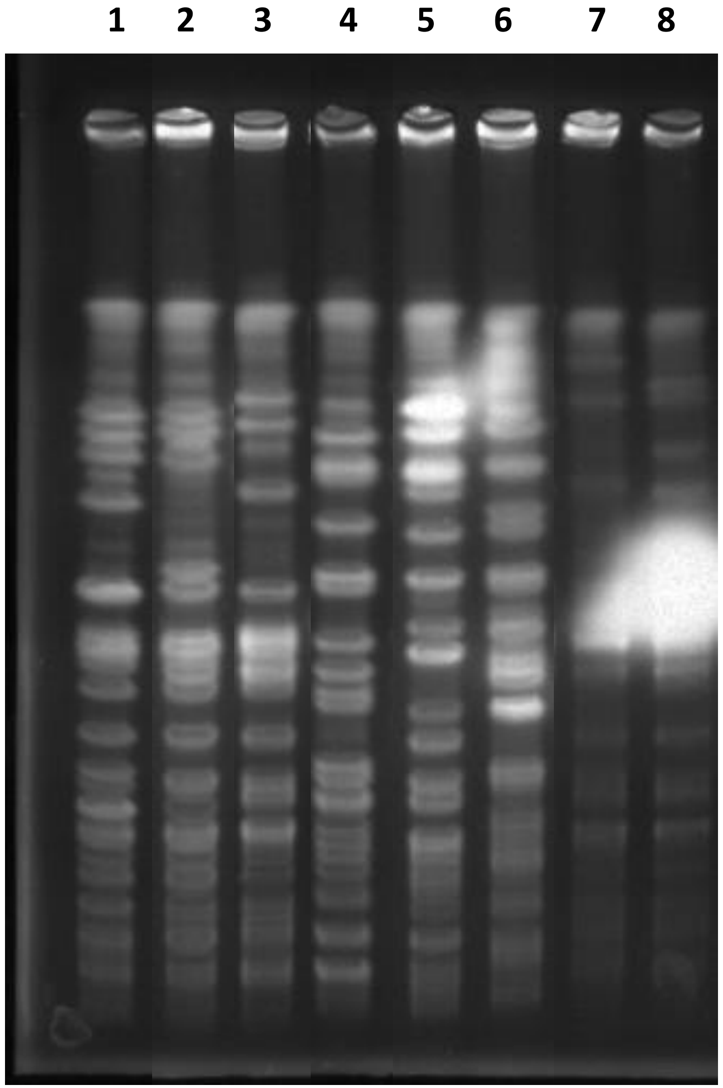

Supplement: Supplementary file 5 — Supplementary file5 (PDF 144 KB) [file 10096_2024_4768_MOESM5_ESM.pdf]
