# Supplementary material for: Genetic analysis of vancomycin-variable Enterococcus faecium clinical isolates in Italy
Source: Eur J Clin Microbiol Infect Dis. 2024 Jan 31;43(4):673–82. doi: 10.1007/s10096-024-04768-0 (PMC10965585; doi:10.1007/s10096-024-04768-0)

Figure S2. Maximum likelihood phylogenetic tree of the 7 VVE*Ita*-S and *E. faecium* 741160 strains. The PFGE pulsotypes and the sequence type (ST) of each isolate are shown. Ef indicate *Enterococcus faecium.*

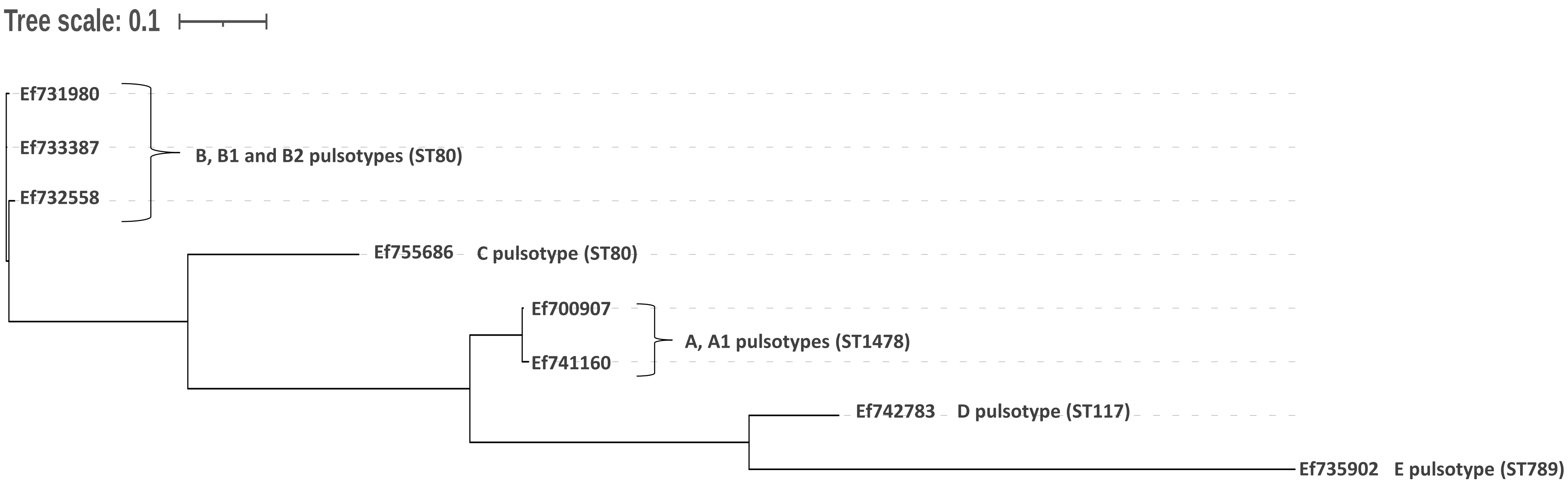

Supplement: Supplementary file 6 — Supplementary file6 (PDF 137 KB) [file 10096_2024_4768_MOESM6_ESM.pdf]
