# Supplementary material for: Genetic analysis of vancomycin-variable Enterococcus faecium clinical isolates in Italy
Source: Eur J Clin Microbiol Infect Dis. 2024 Jan 31;43(4):673–82. doi: 10.1007/s10096-024-04768-0 (PMC10965585; doi:10.1007/s10096-024-04768-0)
Supplement: Supplementary file 7 — Supplementary file7 (PDF 90 KB) [file 10096_2024_4768_MOESM7_ESM.pdf]

Table S1. Single-nucleotide polymorphism (SNPs) of sequenced VVE isolates.

**Isolates**	700907	731980	732558	733387	735902	741160	742783	755686
700907	0	2107	2125	2093	2804	47	1833	2101
731980	2107	0	52	18	3746	2134	2870	1587
732558	2125	52	0	70	3762	2152	2890	1597
733387	2093	18	70	0	3762	2126	2886	1603
735902	2804	3746	3762	3762	0	2831	2378	3454
741160	47	2134	2152	2126	2831	0	1856	2126
742783	1833	2870	2890	2886	2378	1856	0	2716
755686	2101	1587	1597	1603	3454	2126	2716	0

SNPs min: 18 max: 3762
