# Supplementary material for: Genetic analysis of vancomycin-variable Enterococcus faecium clinical isolates in Italy
Source: Eur J Clin Microbiol Infect Dis. 2024 Jan 31;43(4):673–82. doi: 10.1007/s10096-024-04768-0 (PMC10965585; doi:10.1007/s10096-024-04768-0)
Supplement: Supplementary file 8 — Supplementary file8 (DOCX 21 KB) [file 10096_2024_4768_MOESM8_ESM.docx]

**Table S2.** Amino acid sequence identities/similarities of putative proteins encoded by the pEfm700907-vanA (GenBank accession no. OR208591) of the *E. faecium* 700907.

---------------------------------------------------------------------------------------------------------------------------------------------------------------------------------------------------------------------------------------------------- BLASTP analysis*a* Size ----------------------------------------------------------------------------------------------------------------------------------------------------------------------------------------------------

ORF Start Stop (amino Predicted function % Amino acid

(bp) (bp) acids) Most significant database match Accession no. identity (% amino

acid similarity)

----------------------------------------------------------------------------------------------------------------------------------------------------------------------------------------------------------------------------------------------------

*orf1* 1 1041 346 Replication initiation protein Replication protein RepA [*Enterococcus faecium*] BDP48539.1 100 (100)

Δ*orf2* 2250 1657 197 IS6 family transposase IS*6*-like element IS*1216* family transposase, partial [*E. faecium*] WP_080489831.1 99 (99)

*orf3* 2292 2978 228 IS6 family transposase IS6 family transposase [*Enterococcus faecalis*] ARQ19070.1 100 (100)

*orf4* 3736 3026 236 Plasmid replication protein Replication initiation protein [*E. faecium*] HAP9036387.1 99 (99)

*orf5* 5161 5445 94 Hypothetical protein [*E. faecium*] WP_266119626.1 99 (100)

*orf6* 6199 5513 228 IS6 family transposase IS*6* family transposase [*E. faecalis*] EGO8392756.1 100 (100)

*orf7* 6496 7464 322 D-lactate dehydrogenase VanH Vancomycin resistance protein VanH [*E. faecium*] Q05709.1 100 (100)

*orf8* 7457 8488 343 D-alanine--(R)-lactate ligase D-alanine--(R)-lactate ligase VanA [*E. faecium*] HBM8952485.1 99 (100)

*orf9* 8494 9102 202 D-alanyl-D-alanine dipeptidase Vancomycin B-type resistance protein VanX [*E. faecium*] Q06241.1 100 (100)

*orf10* 10130 9222 302 IS*982* family transposase IS*Efm*1, transposase [*E. faecium* Aus0004] AFC62375.1 100 (100)

*orf11* 10578 11489 303 D-Ala-D-Ala dipeptidase/carboxypeptidase DD-carboxypeptidase [*E. faecium*] P37711.1 100 (100)

*orf12* 11642 12127 161 Teicoplanin resistance protein VanZ Protein VanZ [*E. faecium*] Q06242.2 100 (100)

*orf13* 14292 12652 546 Mercuric ion reductase Mercuric reductase MerA [*E. faecium* Com15] EEV63163.1 100 (100)

*orf14* 14704 14306 132 Mercuric resistance regulatory protein, MerR MerR family transcriptional regulator [*E. faecium* Com15] EEV63162.1 100 (100)

*orf15* 15021 15572 183 Tyrosine recombinase Phage integrase [*E. faecium* ATCC 8459] AGE31333.1 100 (100)

*orf16* 15885 16427 180 Hypothetical protein T641_10295 [*E. faecium* MRSN 4777] KKJ72108.1 100 (100)

*orf17* 16938 17228 96 IS*3* family transposase Transposase [*E. faecium*] ALZ53562.1 100 (100)

*orf18* 17264 18100 278 IS*3* family transposase IS*3* family transposase [*E. faecium*] WP_154213969.1 100 (100)

*orf19* 18294 18560 88 YfhO family protein [*Enterococcus faecium*] MBH0800404.1 99 (100)

*orf20* 20757 19462 431 ISEfa5 family transposase ISL3-like element ISEfa5 family transposase [*Enterococcus faecium*] WP_199004470.1 99 (100)

*orf21* 21050 21946 298 ParA family protein [*Bacteria*] WP_002326827.1 100 (100)

*orf22* 22044 22253 69 Transcriptional regulator Omega protein [*Enterococcus faecium*] MBK4807767.1 99 (98)

*orf23* 22271 22543 90 Epsilon antitoxin Antitoxin [*Enterococcus faecium*] WP_104770826.1 99 (100)

*orf24* 22545 23408 287 Zeta toxin Zeta toxin family protein [*Enterococcus faecium*] WP_113827883.1 99 (99)

*orf25* 23965 24651 228 IS6 family transposase IS6-like element IS1216 family transposase [*Enterococcus faecium*] MCZ2247035.1 99 (99)

*orf26* 25162 24674 162 Plasmid replication initiation protein Replication protein Rep [*Enterococcus faecium*] AWB15732.1 97 (99)

*orf27* 26748 26323 141 Hypothetical protein [*Enterococcus faecium*] EGP5549539.1 99 (99)

*orf28* 27822 27406 138 Hypothetical protein [*Enterococcus faecium*] WP_195424410.1 99 (100)

*orf29* 28978 28481 165 DUF536 domain-containing protein [*Enterococcus faecium*] WP_002347002.1 100 (100)

*orf30* 29585 30271 228 IS6 family transposase IS6-like element IS1216 family transposase [*Enterococcus faecium*] MCZ2247035.1 99 (99)

*orf31* 31315 30305 336 Hypothetical protein, partial [*Enterococcus faecium*] MCZ1334167.1 100 (100)

*orf32* 32308 31622 228 IS6 family transposase IS6-like element IS1216 family transposase [*Enterococcus faecium*] MCZ2247035.1 99 (99)

*orf33* 33532 34341 269 Integrase, catalytic region IS30 family transposase [*Enterococcus*] WP_228012590.1 99 (100)

*orf34* 34428 35033 201 Fic domain protein Fic family protein [Enterococcus faecium] WP_139910168.1 99 (100)

*orf35* 35049 35621 109 Site-specific recombinase recombinase family protein [*Enterococcus faecium*] HAQ4760375.1 99 (99)

*orf36* 37013 36054 319 Integrase, catalytic region IS30-like element IS1252 family transposase [*Enterococcus faecium*] MBJ1016605.1 99 (100)

*orf37* 37827 37141 228 IS6 family transposase IS6-like element IS1216 family transposase [*Enterococcus faecium*] MCZ2247035.1 99 (99)

*orf38* 37883 38578 231 Hypothetical protein [*Enterococcus*] WP_002326819.1 100 (100)

*orf39* 39269 39538 89 YefM protein Toxin-antitoxin system Phd/YefM family antitoxin [*Enterococcus faecium*] EGP5080672.1 99 (98)

*orf40* 39531 39788 85 YoeB toxin protein Txe/YoeB family addiction module toxin [*Enterococcus faecium*] MBK4852254.1 100 (100)

*orf41* 40247 41251 334 Hypothetical protein, partial [*Enterococcus faecium*] WP_230853401.1 100 (100)

*orf42* 42030 41416 204 Site-specific recombinase Recombinase family protein [*Bacteria*] WP_001261742.1 100 (100) *orf43* 42480 43805 441 ImpB/MucB/SamB family protein Y-family DNA polymerase [*Enterococcus faecium*] HAQ7475362.1 99 (100)

*orf44* 44460 44750 96 Replication control protein PrgN Type III secretion system protein PrgN [*Enterococcus faecium*] HBD0771398.1 99 (100)

*orf45* 45118 45906 262 Partitioning protein ParA ParA family protein [*Enterococcus faecium*] HAP6146794.1 99 (99)

*orf46* 45890 46219 109 Hypothetical protein, partial [*Enterococcus faecium*] WP_154494709.1 99 (100)

----------------------------------------------------------------------------------------------------------------------------------------------------------------------------------------------------------------------------------------------------

*^a^*For each ORF, only the most significant identity detected is listed
