# Supplementary material for: Genetic analysis of vancomycin-variable Enterococcus faecium clinical isolates in Italy
Source: Eur J Clin Microbiol Infect Dis. 2024 Jan 31;43(4):673–82. doi: 10.1007/s10096-024-04768-0 (PMC10965585; doi:10.1007/s10096-024-04768-0)
Supplement: Supplementary file 9 — Supplementary file9 (DOCX 23 KB) [file 10096_2024_4768_MOESM9_ESM.docx]

**Table S3.** Amino acid sequence identities/similarities of putative proteins encoded by the pEfm731980-vanA (GenBank accession no. OR234011) of the *E. faecium* 731980.

---------------------------------------------------------------------------------------------------------------------------------------------------------------------------------------------------------------------------------------------------- BLASTP analysis*a* Size ----------------------------------------------------------------------------------------------------------------------------------------------------------------------------------------------------

ORF Start Stop (amino Predicted function % Amino acid

(bp) (bp) acids) Most significant database match Accession no. identity (% amino

acid similarity)

----------------------------------------------------------------------------------------------------------------------------------------------------------------------------------------------------------------------------------------------------

*orf1* 1 1041 346 Replication initiation protein Replication protein RepA [*Enterococcus faecium*] BDP48539.1 100 (100)

*orf2* 2343 1657 228 IS6 family transposase IS*6*-like element IS*1216* family transposase [*E. faecium*] KAB7578479.1 99 (100)

*orf3* 3101 2391 236 Plasmid replication protein Replication initiation protein [*E. faecium*] MCZ1195426.1 100 (100)

*orf4* 4556 4750 64 Hypothetical protein [*E. faecium*] WP_107593154.1 100 (100)

*orf5* 5459 4773 228 IS6 family transposase IS*6* family transposase [*E. faecalis*] EGO8392756.1 100 (100)

*orf6* 5756 6724 326 D-lactate dehydrogenase VanH Vancomycin resistance protein VanH [*E. faecium*] ADO66796.1 100 (100)

*orf7* 6717 7748 343 D-alanine--(R)-lactate ligase D-alanine--(R)-lactate ligase VanA [*E. faecium*] HBM8952485.1 99 (100)

*orf8* 7754 8362 202 D-alanyl-D-alanine dipeptidase Vancomycin B-type resistance protein VanX [*E. faecium*] HAQ5904304.1 100 (100)

*orf9* 9390 8482 302 IS*982* family transposase IS*Efm*1, transposase [*E. faecium* Aus0004] WP_113848241.1 100 (100)

*orf10* 9838 10749 303 D-Ala-D-Ala dipeptidase/carboxypeptidase DD-carboxypeptidase [*E. faecium*] ADO66792.1 100 (100)

*orf11* 10902 11387 161 Teicoplanin resistance protein VanZ Protein VanZ [*E. faecium*] AOO95698.1 99 (100)

*orf12* 13552 11912 546 Mercuric ion reductase Mercury(II) reductase [*Enterococcus faecium*] HCD4411652.1 99 (99)

*orf13* 13964 13566 132 Mercuric resistance regulatory protein, MerR MerR family transcriptional regulator [*E. faecium*] WP_264379522.1 99 (100)

*orf14* 14281 14832 183 Tyrosine recombinase Tyrosine-type recombinase/integrase [Enterococcaceae] WP_002307628.1 100 (100)

*orf15* 15145 15687 180 Hypothetical protein [*E. faecium*] MBK4849403.1 99 (100)

*orf16* 16198 16488 96 IS*3* family transposase Transposase [*E. faecium*] ALZ53562.1 100 (100)

*orf17* 16524 17360 278 IS*3* family transposase IS*3* family transposase [*E. faecium*] WP_154213969.1 100 (100)

*orf18* 17554 17820 88 YfhO family protein [*Enterococcus faecium*] MBH0800404.1 99 (100)

*orf19* 20017 18722 431 ISEfa5 family transposase ISL3-like element ISEfa5 family transposase [Enterococcus faecium] WP_151076461.1 99 (100)

*orf20* 20352 21206 298 ParA family protein [*Bacteria*] WP_002326827.1 100 (100)

*orf21* 21304 21513 69 Transcriptional regulator Omega protein [*Enterococcus faecium*] MBK4807767.1 99 (98)

*orf22* 21531 21803 90 Epsilon antitoxin Antitoxin [*Enterococcus faecium*] WP_104770826.1 99 (100)

*orf23* 21805 22668 287 Zeta toxin Zeta toxin family protein [*Enterococcus faecium*] WP_113827883.1 99 (99)

*orf24* 23225 23911 228 IS6 family transposase IS6-like element IS1216 family transposase [*Enterococcus faecium*] MCZ2247035.1 99 (99)

*orf25* 24422 23934 162 Plasmid replication initiation protein Replication protein Rep [*Enterococcus faecium*] AWB15732.1 97 (99)

*∆orf26* 25260 25000 162 Mobilization protein MobC family plasmid mobilization protein [*Enterococcus faecium*] WP_196003520.1 85 (92)

*orf27* 25979 25554 141 Hypothetical protein [*Enterococcus faecium*] EGP5549539.1 99 (99)

*orf28* 27053 26637 138 Hypothetical protein [*Enterococcus faecium*] WP_195424410.1 99 (100)

*orf29* 28209 27712 165 DUF536 domain-containing protein [*Enterococcus faecium*] WP_002347002.1 100 (100)

*orf30* 28816 29502 228 IS6 family transposase IS6-like element IS1216 family transposase [*Enterococcus faecium*] MCZ2247035.1 99 (99)

*orf31* 30546 29536 336 Hypothetical protein, partial [*Enterococcus faecium*] MCZ1334167.1 100 (100)

*orf32* 31539 30853 228 IS6 family transposase IS6-like element IS1216 family transposase [*Enterococcus faecium*] MCZ2247035.1 99 (99)

*orf33* 31595 32299 234 Hypothetical protein [*Enterococcus faecium*] MCZ1768805.1 100 (100)

*orf34* 32763 33572 269 Integrase, catalytic region IS30 family transposase [*Enterococcus*] WP_228012590.1 99 (100)

*orf35* 33659 34264 201 Fic domain protein Fic family protein [*Enterococcus faecium*] WP_139910168.1 99 (100)

*orf36* 34280 34852 109 Site-specific recombinase Recombinase family protein [*Enterococcus faecium*] HAQ4760375.1 99 (99)

*orf37* 36244 35285 319 Integrase, catalytic region IS30-like element IS1252 family transposase [*Enterococcus faecium*] MBJ1016605.1 99 (100)

*orf38* 37058 36372 228 IS6 family transposase IS6-like element IS1216 family transposase [*Enterococcus faecium*] MCZ2247035.1 99 (99)

*orf39* 37114 37809 231 Hypothetical protein [*Enterococcus*] WP_002326819.1 100 (100)

*orf40* 38158 37856 100 Hypothetical protein [*Enterococcus faecium*] AAO52834.1 100 (100)

*orf41* 38574 38978 134 IS200/IS605 family transposase ISEfa4 transposase [*Enterococcus faecium*] WP_079158048.1 99 (100)

*orf42* 38995 40143 382 IS200/IS605 family element RNA-guided endonuclease TnpB [*Bacteria*] WP_002287525.1 100 (100)

*orf43* 40391 40660 89 YefM protein Toxin-antitoxin system Phd/YefM family antitoxin [*Enterococcus faecium*] EGP5080672.1 99 (98)

*orf44* 40653 40910 85 YoeB toxin protein Txe/YoeB family addiction module toxin [*Enterococcus faecium*] MBK4852254.1 100 (100)

*orf45* 41369 42373 334 Hypothetical protein, partial [*Enterococcus faecium*] WP_230853401.1 100 (100)

*orf46* 43152 42538 204 Site-specific recombinase Recombinase family protein [*Bacteria*] WP_001261742.1 100 (100) *orf47* 43602 44927 441 ImpB/MucB/SamB family protein Y-family DNA polymerase [*Enterococcus faecium*] HAQ7475362.1 99 (100)

*orf48* 44920 45270 96 DNA-directed RNA polymerase beta subunit Hypothetical protein U9C_03165 [*Enterococcus faecalis EnGen0253*] EOM19036.1 100 (100)

*orf49* 45582 45872 96 Replication control protein PrgN Type III secretion system protein PrgN [*Enterococcus faecium*] HBD0771398.1 99 (100)

*orf50* 46240 47028 262 Partitioning protein ParA ParA family protein [*Enterococcus faecium*] HAP6146794.1 99 (99)

*orf51* 47015 47341 109 Hypothetical protein, partial [*Enterococcus faecium*] WP_154494709.1 99 (100)

----------------------------------------------------------------------------------------------------------------------------------------------------------------------------------------------------------------------------------------------------

*^a^*For each ORF, only the most significant identity detected is listed
