# Supplementary material for: Genetic analysis of vancomycin-variable Enterococcus faecium clinical isolates in Italy
Source: Eur J Clin Microbiol Infect Dis. 2024 Jan 31;43(4):673–82. doi: 10.1007/s10096-024-04768-0 (PMC10965585; doi:10.1007/s10096-024-04768-0)
Supplement: Supplementary file 10 — Supplementary file10 (DOCX 20 KB) [file 10096_2024_4768_MOESM10_ESM.docx]

**Table S4.** Amino acid sequence identities/similarities of putative proteins encoded by the pEfm732558-vanA (GenBank accession no. OR251469) of the *E. faecium* 732558.

---------------------------------------------------------------------------------------------------------------------------------------------------------------------------------------------------------------------------------------------------- BLASTP analysis*a* Size ----------------------------------------------------------------------------------------------------------------------------------------------------------------------------------------------------

ORF Start Stop (amino Predicted function % Amino acid

(bp) (bp) acids) Most significant database match Accession no. identity (% amino

acid similarity)

----------------------------------------------------------------------------------------------------------------------------------------------------------------------------------------------------------------------------------------------------

Δ*orf1* 1 477 158 Plasmid replication initiation protein Replication initiation protein [Enterococcus faecium] MCZ1337042.1 100 (100)

*orf2* 487 984 165 DUF536 domain-containing protein [Enterococcus faecium] WP_002347002.1 100 (100)

*orf3* 1643 2059 138 Hypothetical protein [Enterococcus faecium] WP_195424410.1 99 (100)

*orf4* 2717 3142 141 Bacteriocin 51 precursor BacA [Enterococcus faecium] WP_224459562.1 99 (100)

*orf5* 3435 3695 86 Mobilization protein MobC family plasmid mobilization relaxosome protein [E. faecium] WP_196003520.1 83 (90)

*orf6* 4294 4782 162 Plasmid replication initiation protein Replication protein Rep [Enterococcus faecium] AWB15732.1 91 (96)

*orf7* 5491 4805 228 IS6 family transposase IS6-like element IS1216 family transposase [*Enterococcus faecium*] MCZ2247035.1 99 (99)

*orf8* 6911 6048 287 Zeta toxin Zeta toxin family protein [*Enterococcus faecium*] WP_113827883.1 99 (99)

*orf9* 7185 6913 90 Epsilon toxin Antitoxin [*Enterococcus faecium*] WP_104770826.1 99 (100)

*orf10* 7412 7203 69 Transcriptional regulator Omega protein [*Enterococcus faecium*] MBK4807767.1 99 (98)

*orf11* 8406 7510 298 ParA family protein [Enterococcus faecalis] WP_089202011.1 99 (100)

*orf12* 8699 9994 431 ISEfa5 family transposase ISL3-like element ISEfa5 family transposase [*Enterococcus faecium*] WP_199004470.1 99 (100)

*orf13* 11162 10896 88 YfhO family protein [*Enterococcus faecium*] MBH0800404.1 99 (100)

*orf14* 12192 11356 278 IS*3* family transposase IS*3* family transposase [*E. faecium*] WP_154213969.1 100 (100)

*orf15* 12518 12228 96 IS*3* family transposase Transposase [*E. faecium*] ALZ53562.1 100 (100)

*orf16* 13571 13029 180 PBECR4 domain-containing protein [Enterococcus faecium] WP_127822045.1 99 (100)

*orf17* 14435 13884 183 Tyrosine recombinase Tyrosine-type recombinase/integrase [Enterococcus faecium] WP_128701000.1 99 (100)

*orf18* 14752 15150 132 Mercuric resistance regulatory protein, MerR MerR family transcriptional regulator [*Bacteria*] WP_002301360.1 100 (100)

*orf19* 15164 16804 546 Mercuric ion reductase Mercury (II) reductase [Enterococcus faecium] EGP5032460.1 99 (100)

*orf20* 17814 17329 161 Teicoplanin resistance protein VanZ Glycopeptide resistance protein VanZ-A [*Bacteria*] WP_000516404.1 100 (100)

*orf21* 18878 17967 303 D-Ala-D-Ala dipeptidase/carboxypeptidase D-Ala-D-Ala carboxypeptidase VanY-A [*Bacteria*] WP_001812592.1 100 (100)

*orf22* 19326 20234 302 IS*982* family transposase IS*982*-like element ISEfm1 family transposase [*Bacteria*] WP_002295743.1 100 (100)

*orf23* 20962 20354 202 D-alanyl-D-alanine dipeptidase D-Ala-D-Ala dipeptidase VanX-A [*Bacteria*] WP_000402347.1 100 (100)

*orf24* 21999 20968 343 D-alanine--(R)-lactate ligase D-alanine--(R)-lactate ligase VanA [*Enterococcus* *faecium*] WP_063856521.1 99 (100)

*orf25* 22960 21992 322 D-lactate dehydrogenase VanH vancomycin resistance protein VanH [Enterococcus faecium] ADO66796.1 100 (100)

*orf26* 23257 23943 228 IS6 family transposase IS6-like element IS1216 family transposase [*Enterococcus faecium*] MCZ2247035.1 99 (100)

*orf27* 25684 26394 236 replication initiation protein [Enterococcus faecium] MCZ1195426.1 100 (100)

*orf28* 26442 27128 228 IS6 family transposase IS6-like element IS1216 family transposase [Enterococcus faecium] MCZ2247035.1 99 (100)

*orf29* 27926 27240 228 IS6 family transposase IS6-like element IS1216 family transposase [Enterococcus faecium] MCZ2247035.1 99 (100)

*orf30* 27982 28686 234 hypothetical protein [Enterococcus faecium] MCZ1768805.1 100 (100)

*orf31* 29150 29959 269 Integrase, catalytic region IS30 family transposase [Enterococcus faecalis] ARQ19074.1 100 (100)

*orf32* 30046 30651 201 Fic domain protein Fic family protein [Enterococcus faecium] WP_139910168.1 99 (100)

*orf33* 30667 31239 190 Site-specific recombinase recombinase family protein [Enterococcus faecium] WP_169038514.1 99 (99)

*orf34* 32631 31672 319 Integrase, catalytic region IS30-like element IS1252 family transposase [*Enterococcus faecium*] MBJ1016605.1 99 (100)

*orf35* 33445 32759 228 IS6 family transposase IS6-like element IS1216 family transposase [Enterococcus faecium] MCZ2247035.1 99 (100)

*orf36* 33501 34196 231 Hypothetical protein [*Enterococcus*] WP_002326819.1 100 (100)

*orf37* 34887 35156 89 YefM protein Toxin-antitoxin system Phd/YefM family antitoxin [*Enterococcus faecium*] WP_129170992.1 99 (100)

*orf38* 35149 35406 85 YoeB toxin protein Txe/YoeB family addiction module toxin [*Enterococcus faecium*] MBK4852254.1 100 (100)

*orf39* 35865 36869 334 Hypothetical protein, partial [Enterococcus faecium] WP_230853401.1 100 (100)

*orf40* 37648 37034 204 Site-specific recombinase Recombinase family protein [*Bacteria*] WP_001261742.1 100 (100)

*orf41* 38098 39423 441 ImpB/MucB/SamB family protein Y-family DNA polymerase [*Enterococcus faecium*] HAQ7475362.1 99 (100)

*orf42* 40078 40368 96 Replication control protein PrgN Type III secretion system protein PrgN [*Enterococcus faecium*] HBD0771398.1 99 (100)

*orrf43* 40736 41524 262 Partitioning protein ParA ParA family protein [*Enterococcus faecium*] HAP6146794.1 99 (99)

*Δorf44* 42186 42770 194 Replication initiation protein A replication initiator protein A [Enterococcus faecium] MCU1867227.1 100 (100)

*Δorf45* 42848 43231 127 Replication-associated protein RepA replication protein RepA, partial [Enterococcus faecium] RCT64424.1 100 (100)

*Δorf46* 44440 43847 197 IS6 family transposase IS6-like element IS1216 family transposase [Enterococcus faecium] WP_085815749.1 99 (99)

*orf47* 45168 44482 228 IS6 family transposase IS6-like element IS1216 family transposase [Enterococcus faecium] MCZ2247035.1 99 (100)

----------------------------------------------------------------------------------------------------------------------------------------------------------------------------------------------------------------------------------------------------

*^a^*For each ORF, only the most significant identity detected is listed
