# Supplementary material for: Genetic analysis of vancomycin-variable Enterococcus faecium clinical isolates in Italy
Source: Eur J Clin Microbiol Infect Dis. 2024 Jan 31;43(4):673–82. doi: 10.1007/s10096-024-04768-0 (PMC10965585; doi:10.1007/s10096-024-04768-0)
Supplement: Supplementary file 11 — Supplementary file11 (DOCX 23 KB) [file 10096_2024_4768_MOESM11_ESM.docx]

**Table S5.** Amino acid sequence identities/similarities of putative proteins encoded by the pEfm733387-vanA (GenBank accession no. OR234012) of the *E. faecium* 733387.

---------------------------------------------------------------------------------------------------------------------------------------------------------------------------------------------------------------------------------------------------- BLASTP analysis*a* Size ----------------------------------------------------------------------------------------------------------------------------------------------------------------------------------------------------

ORF Start Stop (amino Predicted function % Amino acid

(bp) (bp) acids) Most significant database match Accession no. identity (% amino

acid similarity)

----------------------------------------------------------------------------------------------------------------------------------------------------------------------------------------------------------------------------------------------------

*orf1* 1 1041 346 Replication initiation protein Replication protein RepA [*Enterococcus faecium*] BDP48539.1 100 (100)

*orf2* 2343 1657 228 IS6 family transposase IS*6*-like element IS*1216* family transposase [*E. faecium*] KAB7578479.1 99 (100)

*orf3* 2640 3608 326 D-lactate dehydrogenase VanH Vancomycin resistance protein VanH [*E. faecium*] ADO66796.1 100 (100)

*orf4* 3601 4632 343 D-alanine--(R)-lactate ligase D-alanine--(R)-lactate ligase VanA [*E. faecium*] HBM8952485.1 99 (100)

*orf5* 4638 5246 202 D-alanyl-D-alanine dipeptidase Vancomycin B-type resistance protein VanX [*E. faecium*] HAQ5904304.1 100 (100)

*orf6* 6274 5366 302 IS*982* family transposase IS*Efm*1, transposase [*E. faecium* Aus0004] WP_113848241.1 100 (100)

*orf7* 6722 7633 303 D-Ala-D-Ala dipeptidase/carboxypeptidase DD-carboxypeptidase [*E. faecium*] ADO66792.1 100 (100)

*orf8* 7786 8271 161 Teicoplanin resistance protein VanZ Protein VanZ [*E. faecium*] AOO95698.1 99 (100)

*orf9* 10436 8796 546 Mercuric ion reductase Mercury(II) reductase [*Enterococcus faecium*] HCD4411652.1 99 (99)

*orf10* 10848 10450 132 Mercuric resistance regulatory protein, MerR MerR family transcriptional regulator [*E. faecium*] WP_264379522.1 99 (100)

*orf11* 11165 11716 183 Tyrosine recombinase Tyrosine-type recombinase/integrase [*Enterococcaceae*] WP_002307628.1 100 (100)

*orf12* 12029 12571 180 Hypothetical protein [*E. faecium*] MBK4849403.1 99 (100)

*orf13* 13082 13372 96 IS*3* family transposase Transposase [*E. faecium*] ALZ53562.1 100 (100)

*orf14* 13408 14244 278 IS*3* family transposase IS*3* family transposase [*E. faecium*] WP_154213969.1 100 (100)

*orf15* 14438 14704 88 YfhO family protein [*Enterococcus faecium*] MBH0800404.1 99 (100)

*orf16* 14786 15082 98 Hypothetical protein [*E. faecium*] WP_002307630.1 100 (100)

*orf17* 16901 15606 431 ISEfa5 family transposase ISL3-like element ISEfa5 family transposase [*Enterococcus faecium*] WP_151076461.1 99 (100)

*orf18* 17236 18090 298 ParA family protein [*Bacteria*] WP_002326827.1 100 (100)

*orf19* 18188 18397 69 Transcriptional regulator Omega protein [*Enterococcus faecium*] MBK4807767.1 99 (98)

*orf20* 18415 18687 90 Epsilon antitoxin Antitoxin [*Enterococcus faecium*] WP_104770826.1 99 (100)

*orf21* 18689 19552 287 Zeta toxin Zeta toxin family protein [*Enterococcus faecium*] WP_113827883.1 99 (99)

*orf22* 20109 20795 228 IS6 family transposase IS6-like element IS1216 family transposase [*Enterococcus faecium*] MCZ2247035.1 99 (99)

*orf23* 21306 20818 162 Plasmid replication initiation protein Replication protein Rep [*Enterococcus faecium*] AWB15732.1 97 (99)

∆*orf24* 22197 21904 97 Mobilization protein MobC family plasmid mobilization protein [*Enterococcus faecium*] WP_196003520.1 85 (92)

*orf25* 22882 22457 141 Hypothetical protein [*Enterococcus faecium*] EGP5549539.1 99 (99)

*orf26* 23956 23540 138 Hypothetical protein [*Enterococcus faecium*] WP_195424410.1 99 (100)

*orf27* 25112 24615 165 DUF536 domain-containing protein [*Enterococcus faecium*] WP_002347002.1 100 (100)

*orf28* 25719 26405 228 IS6 family transposase IS6-like element IS1216 family transposase [*Enterococcus faecium*] MCZ2247035.1 99 (99)

*orf29* 27449 26439 336 Hypothetical protein, partial [*Enterococcus faecium*] MCZ1334167.1 100 (100)

*orf30* 28442 27756 228 IS6 family transposase IS6-like element IS1216 family transposase [*Enterococcus faecium*] MCZ2247035.1 99 (99)

*orf31* 28498 29202 234 Hypothetical protein [*Enterococcus faecium*] MCZ1768805.1 100 (100)

*orf32* 29666 30475 269 Integrase, catalytic region IS30 family transposase [*Enterococcus*] WP_228012590.1 99 (100)

*orf33* 30562 31167 201 Fic domain protein Fic family protein [*Enterococcus faecium*] WP_139910168.1 99 (100)

*orf34* 31183 31755 109 Site-specific recombinase Recombinase family protein [*Enterococcus faecium*] HAQ4760375.1 99 (99)

*orf35* 33147 32188 319 Integrase, catalytic region IS30-like element IS1252 family transposase [*Enterococcus faecium*] MBJ1016605.1 99 (100)

*orf36* 33961 33275 228 IS6 family transposase IS6-like element IS1216 family transposase [*Enterococcus faecium*] MCZ2247035.1 99 (99)

*orf37* 34017 34712 231 Hypothetical protein [*Enterococcus*] WP_002326819.1 100 (100)

*orf38* 35061 34759 100 Hypothetical protein [*Enterococcus faecium*] AAO52834.1 100 (100)

*orf39* 35477 35881 134 IS200/IS605 family transposase ISEfa4 transposase [*Enterococcus faecium*] WP_079158048.1 99 (100)

*orf40* 35898 37046 382 IS200/IS605 family element RNA-guided endonuclease TnpB [*Bacteria*] WP_002287525.1 100 (100)

*orf41* 37294 37563 89 YefM protein Toxin-antitoxin system Phd/YefM family antitoxin [*Enterococcus faecium*] EGP5080672.1 99 (98)

*orf42* 37556 37813 85 YoeB toxin protein Txe/YoeB family addiction module toxin [*Enterococcus faecium*] MBK4852254.1 100 (100)

*orf43* 38272 39276 334 Hypothetical protein, partial [*Enterococcus faecium*] WP_230853401.1 100 (100)

*orf44* 40055 39441 204 Site-specific recombinase Recombinase family protein [*Bacteria*] WP_001261742.1 100 (100)

*orf45* 40505 41830 441 ImpB/MucB/SamB family protein Y-family DNA polymerase [*Enterococcus faecium*] HAQ7475362.1 99 (100)

*orf46* 41823 42173 96 DNA-directed RNA polymerase beta subunit Hypothetical protein U9C_03165 [*Enterococcus faecalis EnGen0253*] EOM19036.1 100 (100)

*orf47* 42485 42775 96 Replication control protein PrgN Type III secretion system protein PrgN [*Enterococcus faecium*] HBD0771398.1 99 (100)

*orf48* 43143 43931 262 Partitioning protein ParA ParA family protein [*Enterococcus faecium*] HAP6146794.1 99 (99)

*orf49* 43918 44244 109 Hypothetical protein, partial [*Enterococcus faecium*] WP_154494709.1 99 (100)

----------------------------------------------------------------------------------------------------------------------------------------------------------------------------------------------------------------------------------------------------

*^a^*For each ORF, only the most significant identity detected is listed
